# Reprogramming of arachidonate metabolism confers temozolomide resistance to glioblastoma through enhancing mitochondrial activity in fatty acid oxidation

**DOI:** 10.1186/s12929-022-00804-3

**Published:** 2022-03-25

**Authors:** Yu-Ting Tsai, Wei-Lun Lo, Pin-Yuan Chen, Chiung-Yuan Ko, Jian-Ying Chuang, Tzu-Jen Kao, Wen-Bing Yang, Kwang-Yu Chang, Chia-Yang Hung, Ushio Kikkawa, Wen-Chang Chang, Tsung-I. Hsu

**Affiliations:** 1 Graduate Institute of Medical Sciences, College of Medicine, Taipei Medical University, 250 Wu-Hsing Street, Taipei, Taiwan 110; 2 Department of Surgery, School of Medicine, College of Medicine, Taipei Medical University, Taipei, 110 Taiwan; 3 Department of Neurosurgery, Shuang Ho Hospital, Taipei Medical University, Taipei, 110 Taiwan; 4 TMU Research Center of Neuroscience, Taipei Medical University, 250 Wu-Hsing Street, Taipei, Taiwan; 5 School of Medicine, Chang Gung University, Taoyuan City, 33302 Taiwan; 6 Department of Neurosurgery, Keelung Chang Gung Memorial Hospital, Keelung, 204 Taiwan; 7 Department of Neurosurgery, Linkou Chang Gung Memorial Hospital, Taoyuan, 333 Taiwan; 8 Graduate Institute of Neural Regenerative Medicine, College of Medical Science and Technology, Taipei Medical University, Taipei, 110 Taiwan; 9 Ph.D. Program for Neural Regenerative Medicine, College of Medical Science and Technology, Taipei Medical University and National Health Research Institutes, Taipei, 110 Taiwan; 10 Ph.D. Program in Medical Neuroscience, College of Medical Science and Technology, Taipei Medical University and National Health Research Institutes, Taipei, 110 Taiwan; 11 TMU Research Center of Cancer Translational Medicine, Taipei, 110 Taiwan; 12 National Institute of Cancer Research, National Health Research Institutes, Tainan, 704 Taiwan; 13 Department of Immuno-Oncology, Beckman Research Institute, City of Hope, Duarte, CA 91010 USA

**Keywords:** TMZ-resistant GBM, Sp1, PGE2, Mitochondria, Fatty acid β-oxidation

## Abstract

**Background:**

Sp1 is involved in the recurrence of glioblastoma (GBM) due to the acquirement of resistance to temozolomide (TMZ). Particularly, the role of Sp1 in metabolic reprogramming for drug resistance remains unknown.

**Methods:**

RNA-Seq and mass spectrometry were used to analyze gene expression and metabolites amounts in paired GBM specimens (primary vs. recurrent) and in paired GBM cells (sensitive vs. resistant). ω-3/6 fatty acid and arachidonic acid (AA) metabolism in GBM patients were analyzed by targeted metabolome. Mitochondrial functions were determined by Seahorse XF Mito Stress Test, RNA-Seq, metabolome and substrate utilization for producing ATP. Therapeutic options targeting prostaglandin (PG) E2 in TMZ-resistant GBM were validated in vitro and in vivo.

**Results:**

Among the metabolic pathways, Sp1 increased the prostaglandin-endoperoxide synthase 2 expression and PGE2 production in TMZ-resistant GBM. Mitochondrial genes and metabolites were obviously increased by PGE2, and these characteristics were required for developing resistance in GBM cells. For inducing TMZ resistance, PGE2 activated mitochondrial functions, including fatty acid β-oxidation (FAO) and tricarboxylic acid (TCA) cycle progression, through PGE2 receptors, E-type prostanoid (EP)1 and EP3. Additionally, EP1 antagonist ONO-8713 inhibited the survival of TMZ-resistant GBM synergistically with TMZ.

**Conclusion:**

Sp1-regulated PGE2 production activates FAO and TCA cycle in mitochondria, through EP1 and EP3 receptors, resulting in TMZ resistance in GBM. These results will provide us a new strategy to attenuate drug resistance or to re-sensitize recurred GBM.

**Supplementary Information:**

The online version contains supplementary material available at 10.1186/s12929-022-00804-3.

## Background

Glioblastoma (GBM), which is classified as a grade IV glioma, is the most aggressive and malignant brain tumor [1]. Its current standard treatment is tumour resection followed by chemotherapy and radiotherapy, and in this regard, temozolomide (TMZ), an oral alkylating agent, is the most widely used chemotherapy agent. However, with this current standard treatment, GBM still remains incurable and is characterized by a high prevalence of recurrence owing to its acquisition TMZ resistance [1].

Specificity protein 1 (Sp1), a transcription factor recognizing GC-rich DNA promoter, is overexpressed in many cancers such as breast, gastric, pancreatic, lung, brain, and thyroid cancers to promote tumor initiation and tumor growth [2]. Moreover, Sp1 regulates the DNA damage response, cell apoptosis, cell senescence, the ability to escape from immune system, and tumor angiogenesis, which leads to the cancer drug resistance [2]. Previously, we reported that Sp1 promotes the development of malignant characteristics in GBM, including drug resistance and cancer stem cell enrichment [3–9]. Particularly, it enhances resistance to TMZ by upregulating the expression of cytochrome p450 (CYP) 17A1, which catalyses the metabolism of cholesterol to neurosteroids [5, 6, 10, 11]. Therefore, these findings suggest that Sp1 plays an important role in the acquisition of drug resistance in cancers by regulating the reprogramming of arachidonate metabolism. Therefore, in this study, our objective was to clarify the role of Sp1 in regulating arachidonate metabolism, leading to GBM drug resistance.

With respect to phospholipid-prostaglandin metabolism, prostaglandin E2 (PGE2) is upregulated and is frequently observed in various types of cancer [12–14]. Specifically, in GBM, the enzyme that is primarily responsible for PGE2 synthesis, cyclooxygenase-2 (COX2)/prostaglandin-endoperoxide synthase 2 (PTGS2), reportedly promotes cancer cell proliferation and migration [15, 16], suggesting that COX2 enhances arachidonic acid (AA) metabolism, which is necessary for the survival of GBM cells, to synthesize PGE2. However, the correlation between AA-derived metabolites and drug resistance in GBM remains unclear.

To sustain rapid proliferation, cancer cells take advantage of the reprograming of metabolic networks to generate sufficient bioenergy to support their cellular functions. Even though it is well known that the Warburg effect is the metabolic reprogramming that enhances aerobic glycolysis for cancer proliferation [17], the importance of mitochondria-mediated metabolism has been raised recently to challenge the Warburg effect in GBM. Evidence suggests that GBM cells rely on oxidative phosphorylation in mitochondria to generate bioenergy [18, 19]. Additionally, fatty acid β-oxidation (FAO), which is the main step in lipid metabolism for the generation of bioenergy inside the mitochondria, has also been considered to play an important role in tumour development in nutrient-deprived environments [19] and cancer metastasis [20, 21]. However, its role in cancer drug resistance is still unknown.

In this study, we observed that Sp1 enhances the synthesis of PGE2 from AA in patients with recurrent GBM, and that PGE2 induces TMZ resistance by enhancing mitochondrial activity. Our findings provide a novel mechanism for the reprograming of AA metabolism to the end of combating drug resistance. They also suggest the possibility of realizing GBM treatment based on the combination of the EP1 antagonist, ONO-8713, and TMZ to reverse drug resistance.

## Methods

### Study design and overview

Experimental RNA-Seq and mass spectrometry were used to analyse gene expression and metabolite amounts in paired GBM specimens (primary vs. recurrent) and in paired GBM cells (sensitive vs. resistant). ω-3/6 fatty acid and AA metabolism in GBM patients was analysed by targeted metabolome. Mitochondrial functions in GBM cells were determined by Seahorse XF Mito Stress Test, RNA-Seq, metabolome, and substrate utilization for ATP production. Therapeutic options targeting PGE2 in TMZ-resistant GBM were validated using GBM cells and xenograft animal model.

### Human samples

GBM samples were obtained from patients with GBM admitted to the Keelung Chang Gung Memorial Hospital, Linkou Chang Gung Memorial Hospital, and Taipei Medical University-Shuang-Ho Hospital. All patients received the TMZ-mediated chemotherapy after tumor resection. The characteristics of these patients are summarized in Additional file 1: Tables S1 and S2 and Fig. S1. The pathologies of the human brain tumour samples were determined according to WHO classification [22, 23]. Isocitrate dehydrogenase 1 (IDH-1), glial fibrillary acidic protein (GFAP), and O^6^-methylguanine-DNA methyltransferase (MGMT) were used as the diagnostic and prognostic makers for GBM [24, 25].

### Cell culture

GBM cell lines U87MG, A172, and T98G were purchased from American Type Culture Collection (ATCC, Manassas, VA, USA). Further, Pt#3 and Pt#5 were isolated from patients with GBM, and P1S was obtained from another patient, who exhibited therapeutic resistance right from the onset of treatment [6]. The cells were cultured in Dulbecco’s Modified Eagle Medium (DMEM, Thermo Fisher Scientific, Waltham, MA, USA) supplemented with 10% foetal bovine serum (GE Healthcare Life Sciences, South Logan, UT, USA), 100 μg/mL penicillin, and 100 μg/mL streptomycin (Thermo Fisher Scientific) at 37 ºC in a 5%-CO_2_ incubator. TMZ-resistant U87MG-R and Pt#3-R cells were established via long-term treatment with TMZ as described previously [3, 5].

### Establishment of Sp1 knockout cells

Sp1 knockout cells were prepared using clustered regularly interspaced short palindromic repeats (CRISPR) strategy under the assistance of Biotools Co., Ltd. (New Taipei City, Taiwan). Expression vectors for guide RNA (U6-gRNA) and Cas9 gene (CMV-p-Cas9), which are ampicillin-resistant, were obtained using the Escherichia coli strain DH5α as a host. Surrogate reporter vectors, which are kanamycin resistant, were also purified from the transformed E. coli. Cells were transfected with plasmids by using Lipofectamine® LTX & PLUS™ Reagent (15,338–100, Thermo Fisher Scientific). Briefly, 10 μL of LTX Reagent were diluted in 100 μL of Opti-MEM Medium (Thermo Fisher Scientific), and 2.5 μg of plasmid DNA (Cas9: sgRNA: surrogate = 1:1:0.5) were diluted in 100 μL of Opti-MEM Medium. sgRNA was designed: 5′-AGGAGTTGGTGGCAATAATGGGG-3′. After hygromycin selection, Sp1-knockout cell colonies were identified and the depletion was confirmed by Western blotting.

### Chemicals

Chemicals in Additional file 1: Table S3 were dissolved in the appropriate solvent according to the manufacturers’ instruction for stock solutions. Other materials were described in the respective paragraphs in this section. EP1, EP3, and EP4 antagonists were provided by Ono Pharmaceutical Co., Ltd (Osaka, Japan).

### Chemicals and reagents for targeted metabolomics

All eicosanoids and deuterated IS were purchased from Cayman Chemical (Ann Arbor, MI, USA). HPLC-grade acetonitrile (ACN) and methanol (MeOH) were purchased from Merck (Darmstadt, Germany). MilliQ water (Millipore, Bradford, USA) was used in all experiments. Acetic acid was purchased from Sigma–Aldrich. CNW Poly-Sery MAX SPE cartridges were from ANPEL Co. (Shanghai, PRC). The stock solutions of standards were prepared at the concentration of 0.1 mg/mL in MeOH. All stock solutions were stored at −20 °C. The stock solutions were diluted with MeOH to working solutions before analysis.

### Sample preparation for targeted metabolomics

Tissue samples (about 50 mg) and 10 μL of BHT (butylated hydroxytoluene)/MeOH solution (W/V, 4.8 g/100 mL) were subjected to protein precipitation by adding 100 μL of MeOH containing deuterium-labeled IS, at a final concentration of 50 ng/mL each of PGE2-d4, 6-keto PGF1α-d4, 5-HETE-d8 and 100 ng/mL of 9-HODE-d4. Samples were extracted with 500 μL of MeOH using a tissuelyzer at 50 Hz for 30 s (3 times) followed with 5 ultrasonication cycles (1 min treatment and 1 min break). The supernatant was transferred to new tubes after centrifuged at 12,000 rpm for 10 min at 4 °C, and followed by diluting with pure water to 15% MeOH concentration, followed by solid phase extraction (SPE) pretreated with MeOH and equilibrated with H2O. The extract was dried and then re-dissolved in 100 μL of MeOH, followed by filtering the solution with a 0.22 μm membrane filter before UPLC-MS/MS analysis.

### Liquid chromatography and mass spectrometry

UPLC-MS/MS analyses were conducted on an Agilent UPLC-MS/MS system consisting of 1290 UPLC-system coupled with an Agilent 6470 triple-quadrupole mass spectrometer (Agilent Technologies). For analysis, 3 μL of the extract were injected. Chromatographic separation was achieved on an Agilent ZORBAX RRHD Eclipse XDB C18 column (2.1 × 100 mm, 1.8 μm particles) using a flow rate of 0.659 mL/min at 45 °C during a 13 min gradient (0–12 min from 68% A to 20% A, 12–13 min 5%A), while using the solvents A, water containing 0.005% formic acid, and B, acetonitrile containing 0.005% formic acid. Electrospray ionization was performed in the negative ion mode using N2 at a pressure of 30 psi for the nebulizer with a flow of 10 L/min and a temperature of 300 °C, respectively. The sheath gas temperature was 350 °C with a flow rate of 11 L/min. The capillary was set at 3500 V and the nozzle voltage was 500 V. Multiple reaction monitoring (MRM) has been used for quantification of screening fragment ions.

Data preprocessing: Peak determination and peak area integration were performed with MassHunter Workstation software (Version B.08.00, Agilent Technologies) while auto-integration was manually inspected and corrected if necessary. The obtained peak areas of targets were corrected by appropriate IS and calculated response ratios were used throughout the analysis.

### Untargeted metabolomics

The ultra-high performance liquid chromatography-quadrupole time-of-flight mass spectrometry (UHPLC-Q-TOF–MS) analysis was performed using an UHPLC system (1290, Agilent Technologies, Santa Clara, CA, USA) with a UPLC BEH Amide column (1.7 μm 2.1*100 mm, Waters, Milford, MA, USA) coupled to TripleTOF 6600 (Q-TOF, AB Sciex, Framingham, MA, USA). The mobile phase consisted of 25 mM NH4OAc and 25 mM NH4OH in water(pH = 9.75) (A) and acetonitrile (B) were carried with elution gradient as follows: 0 min, 95% B; 7 min, 65% B; 9 min, 40% B; 9.1 min, 95% B; 12 min, 95% B, which was delivered at 0.5 ml per min. The injection volume was 2 μL. The Triple TOF mass spectrometer was used for its ability to acquire MS/MS spectra on an information dependent basis (IDA) during an LC/MS experiment. In this mode, the acquisition software (Analyst TF 1.7, AB Sciex) continuously evaluates the full scan survey MS data as it collects and triggers the acquisition of MS/MS spectra depending on preselected criteria. In each cycle, 12 precursor ions whose intensity greater than 100 were chosen for fragmentation at collision energy (CE) of 30 V (15 MS/MS events with product ion accumulation time of 50 ms each). ESI source conditions were set as following: Ion source gas 1 as 60 Psi, Ion source gas 2 as 60 Psi, Curtain gas as 35 Psi, source temperature 650℃, Ion Spray Voltage Floating (ISVF) 5000 V or -4000 V in positive or negative modes, respectively.

### Enzyme-linked immunosorbent assay (ELISA)

AA and PGE2 in culture media of wild type and TMZ-resistant U87MG cells were determined using Arachidonic Acid ELISA Kit (E4602, Biovision, Milpitas, CA, USA) and Prostaglandin E2 ELISA Kit (514,010, Cayman, Ann Arbor, MI, USA), respectively, according to the manufacturers’ instruction.

### Promoter reporter assay

Plasmids containing each promoter region were transfected into targeted cell lines. Cells were harvested with diluted Cell Culture lysis 5X Reagent (Promega, San Luis Obispo, CA, USA). 10 μL of sample were then mixed with 10 μL of luciferin (Promega). Luminometer (HIDEX, Tampa, FL, USA) was used to measure the promoter activity of the mixture.

### 3- (4,5-dimethylthiazol-2-yl)-2,5-diphenyltetrazolium bromide (MTT) assay

Cells (2 × 10^4^ cells/well in a 24-well plate) were treated with different doses of drugs for 4 days. After the treatment, 300 μL of fresh medium containing 0.5 mg/mL MTT reagent (Sigma-Aldrich, St. Louis, MO, USA) were added to each well and incubated for 15 min at 37 °C. Medium was removed and the crystals were dissolved in 300 μL of DMSO (Sigma-Aldrich). The absorbance was measured at 550 nm by using an iMark Microplate Absorbance Reader (Bio-Rad, Hercules, CA, USA).

### RNA-Seq and bioinformatics

After total RNA extraction, samples were subjected to genomic sequencing. The gene expression influenced by Sp1 for 1.5 folds was sorted, and the functional grouping was performed using the Ingenuity Pathway Analysis (IPA) system (https://www.qiagenbioinformatics.com/products/ingenuitypathway-analysis). Metabolism genes were sorted using Cancer Cell Metabolism Genes [26]. Heatmaps were prepared based on the level of expression using ToppCluster (https://toppcluster.cchmc.org/).

### Chromatin immunoprecipitation coupled with sequencing (ChIP-Seq)

U87MG cells were fixed with 1% formaldehyde for preserving the protein-DNA interactions, and DNA–protein complexes were harvested using the Simple ChIP enzymatic chromatin IP kit (#9003, Cell Signaling Technology, Danvers, MA, USA) followed by NextSeq 500 high throughput sequencing system (Illumina, San Diego, CA, USA) as described previously [8, 27].

### Determination of mitochondrial morphologies

After transfection with a pDsRed-mito vector (Takara Bio Inc., Shiga, Japan) for 24 h, cells were immune-stained using the DsRed-Monomer antibody (OriGene Technologies, Inc., Rockville, MD, USA) followed by observation under the immunofluorescent microscope. Quantification for fusion / fission was performed according the report [28].

### XFe24 seahorse mitochondrial respiration mito stress test

Cells were treated with different conditions of TMZ, PGE2 or EP1-EP4 antagonists for 4 days. After the treatment, cells were trypsinized and 2 × 10^4^ cells/well were seeded into the XFe24 Cell Culture Microplates (Agilent Technologies) and incubated for a day. Meanwhile, a sensor cartridge (detecting probes, Agilent Technologies) in Seahorse XF Calibrant at 37 °C was hydrated in a non-CO2 incubator overnight for the following experiments. In the assay day, the cell-cultured medium was replaced with assay medium (DMEM without sodium bicarbonate, supplemented with 2% FBS and Penicillin/Streptomycin, pH: 7.4) and incubated for 1 h. Cells were then incubated at 37 °C in a non-CO2 incubator that ready for experiments. Oligomycin (10 μM), carbonyl cyanide-p-trifluoromethoxyphenylhydrazone (FCCP, 2 μM), and rotenone/antimycin A (5 μM) were prepared and placed into the sensor cartridge for the injection in the running procedure. The procedure of the assay was performed according to the guidelines from the XFe24 Seahorse Mitochondrial Respiration Mito Stress Test (Agilent technologies) [29]. For the evaluations of FAO percentage, etomoxir (40 μM) were added into the medium, 90 min before running the assay. FAO-dependent oxygen consumption was calculated as [OCR from groups without etomoxir – OCR from groups treated with etomoxir].

### ATP assay

ATP Colorimetric/Fluorometric Assay kit (K354-100, BioVision, CA, USA) was used to estimate total intracellular ATP amount in cells. After treatment, 10^6^ cells were lysed by 100 μL of ATP assay buffer and incubated for 15 min on ice. Forty μL of cell lysates were mixed with 50 μL by ATP assay buffer and then incubated with the reaction mix (ATP assay buffer 44 μL, ATP probe 2 μL, ATP converter 2 μL, and developer 2 μL) for 30 min, protected from light. The absorbance was measured by OD 570 nm (550 nm) in a microplate reader.

### Plasmids and transfection

GFP-Sp1 and PTGES2 plasmids (HG19428-ACG, Sino Biological, Wayne, PA, USA) were transfected into cells with Poly-Jet™ Reagent (SignaGen Laboratories, Rockvillie, MD, USA) for overexpression. Sp1, PTGS2 and CPT1A siRNAs (Dharmacon, Lafayette, CO, USA) were transfected into cells with Lipofectamine RNAiMAX Reagent (Thermo Fisher Scientific) for knockdown. PLA2G5, ABHD8, and PTGS2 promoter were designed as a 1000 bp sequence before the coding region. The information of the constructs was listed in Additional file 1: Table S4.

### Western blotting

Protein samples were separated on SDS-PAGE and transferred onto the PVDF membrane (Bio-Rad). The PVDF membrane was blocked in 5% nonfat milk in TBST buffer at room temperature for an hour, and then incubated with specific primary antibodies (Additional file 1: Table S5) at 4 °C overnight. After washing with TBST buffer, the membranes were incubated with the appropriate secondary antibodies for another one hour. Finally, the membranes were washed, and then developed by using T-Pro LumiLong Plus Chemiluminescent detection kit (T-Pro Biotechnology, New Taipei City, Taiwan).

### Real-time PCR

The RNA sample was extracted by TRIzol (Thermo Fisher Scientific), and 1 μg of total RNA was subjected to real-time PCR reagent using Prime Script™ RT Reagent kit (Takara Bio. Inc, Shiga, Japan). The expression of each mRNA was determined using 2 × SYBR real time master mix (AB Sciex) and the specific primers (Additional file 1: Table S6). GAPDH was used as the internal control. SYBR green fluorescence was then monitored using an ABI 7000 Sequence Detection System (AB Sciex).

### MitoPlates analysis for estimating the consumption of NADH/FADH-producing substrate

MitoPlates S-1 (Biolog, Hayward, CA, USA) were used according to the manufacturer’s instruction. To dissolve the substrates coated on MitoPlates, assay mixture containing saponin (30 μg/mL) was added into each well and the plate was incubated at 37 °C for 1 h. After the treatment with PGE2, cells were trypsinized and 1.5 × 10^6^ cells were mixed with 1X Mitochondrial Assay Solution (MAS) and added equally into each well. The mixtures were incubated at 37 °C for 2 h and the absorbance was detected at 590 nm.

### Wound-healing migration and Transwell invasion assays

After treatment with ONO-8713 or celecoxib for 48 h, 6 × 10^4^ U87MG-R and Pt#3-R were seeded into Culture-Inserts 2 wells (ibidi GmbH, Grafelfing, Germany) and incubated overnight for the wound-healing assay. Images were taken at the cell wound after removing the insert and incubating for 24 h. The migratory area was quantified via the Image J software. For the Transwell invasion assay, inserts with 8-μm pore (Corning Incorporated, Corning, NY, USA) were coated with matrigel matrix (Corning Incorporated). Subsequently, 2.5 × 10^4^ U87MG-R and Pt#3-R cells were seeded into inserts and incubated for 24 h. Invaded cells on the Transwell membrane were stained with 0.5% crystal violet. Images were quantified via the Image J software.

### Xenograft animal model

TMZ-resistant U87MG-R (1 × 10^6^ cells in 50 μL of DMEM) was injected into the back of 8-week-old CAnN.Cg-Foxn1nu/CrlBltw (BALB/c nude) male mice. Pt#3-R cells (1 × 10^6^ cells in 50 μL of DMEM) were injected into the back of 8-week-old NOD.CB17-Prkdcscid/NcrCrl (NOD/SCID) male mice. A month after transplantation, mice were administrated with TMZ (10 mg/kg), ONO-8713 (15 mg/kg), and celecoxib (10 mg/kg) twice a week by intraperitoneal injection for another 8 weeks. After the treatment, the mice were then sacrificed and the tumors were excised by surgery to measure the weight and size. Tumor size was calculated according to the formula: 1/2 * long side * (short side)^2^.

### Statistical analysis

The data obtained were represented as means ± S.E.M. Two-tailed unpaired Student’s t-test or two-way ANOVA (animal experiments) were used to analyse the differences between the control and experimental groups. *P < 0.05, **P < 0.01, and ***P < 0.001 were considered significant in all comparisons.

## Results

### Importance of Sp1 in phospholipid metabolism to generate AA in TMZ-resistant GBM

**Fig. 1:**
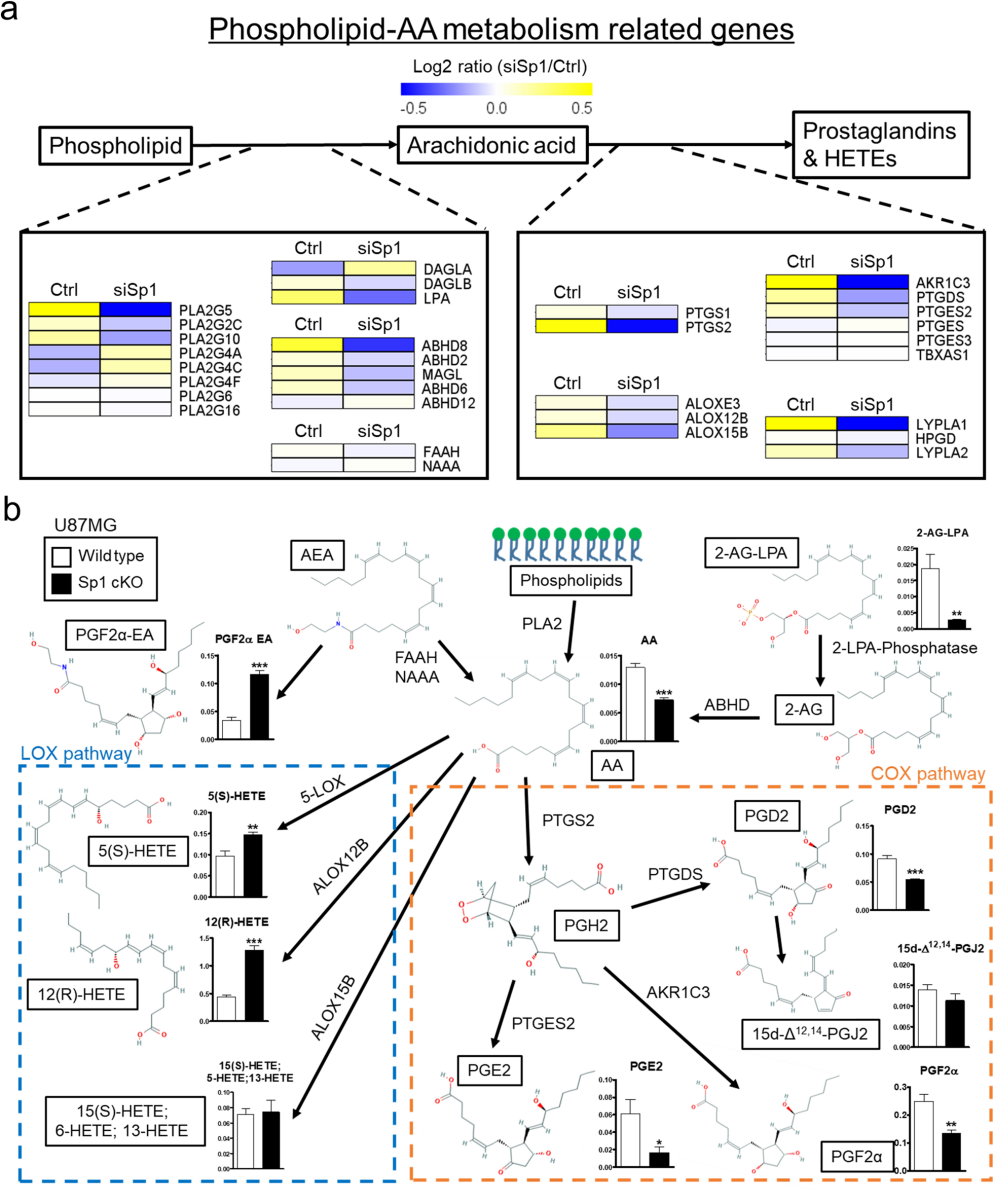
Regulation of phospholipid and AA metabolism by Sp1. **a** Sp1-regulated metabolic genes. After Sp1 knockdown by siRNA, U87MG cell RNA was collected and subjected to RNA-Seq. **b** AA-related metabolite alteration determined via UHPLC-QTOF-MS. U87MG and U87MG-Sp1 cKO cells were subjected to mass spectrometry-based metabolomics analysis. The alteration of metabolite levels was illustrated by performing two-tailed unpaired Student’s t test. The chemical structures were obtained from PubChem, National Library of Medicine. The complete definitions of the different abbreviations are provided in Additional file 1: Table S5

Metabolic reprogramming is critical for cancer progression [30]. In this study, we attempted to elucidate whether Sp1 is involved in metabolic reprogramming during the process of TMZ resistance acquisition in GBM. By analysing the RNA-Seq-based transcriptome data, it was observed that Sp1 knockdown downregulated the expression of phospholipid metabolic-related genes, including phospholipase A2 group V (PLA2G5), abhydrolase domain containing 8 (ABHD8), prostaglandin-endoperoxide synthase 2 (PTGS2)/cyclooxygenase 2 (COX2), prostaglandin E synthase 2 (PTGES2), prostaglandin D synthase (PTGDS), and aldo–keto reductase family 1 member C3 (AKR1C3) (Fig. 1a). Further, Sp1 knockout resulted in a significant decrease in the levels of several AA-derived metabolites, including PGE2, PGD2, and PGF2α; whereas the levels of 5(S)-HETE and 12(R)-HETE were upregulated (Fig. 1b). Overall, these results suggested that Sp1-regulated AA metabolism participates in the acquisition of TMZ resistance in GBM via the COX pathway.

### Importance of Sp1-regulated AA metabolism in TMZ-resistance acquisition in GBM

**Fig. 2:**
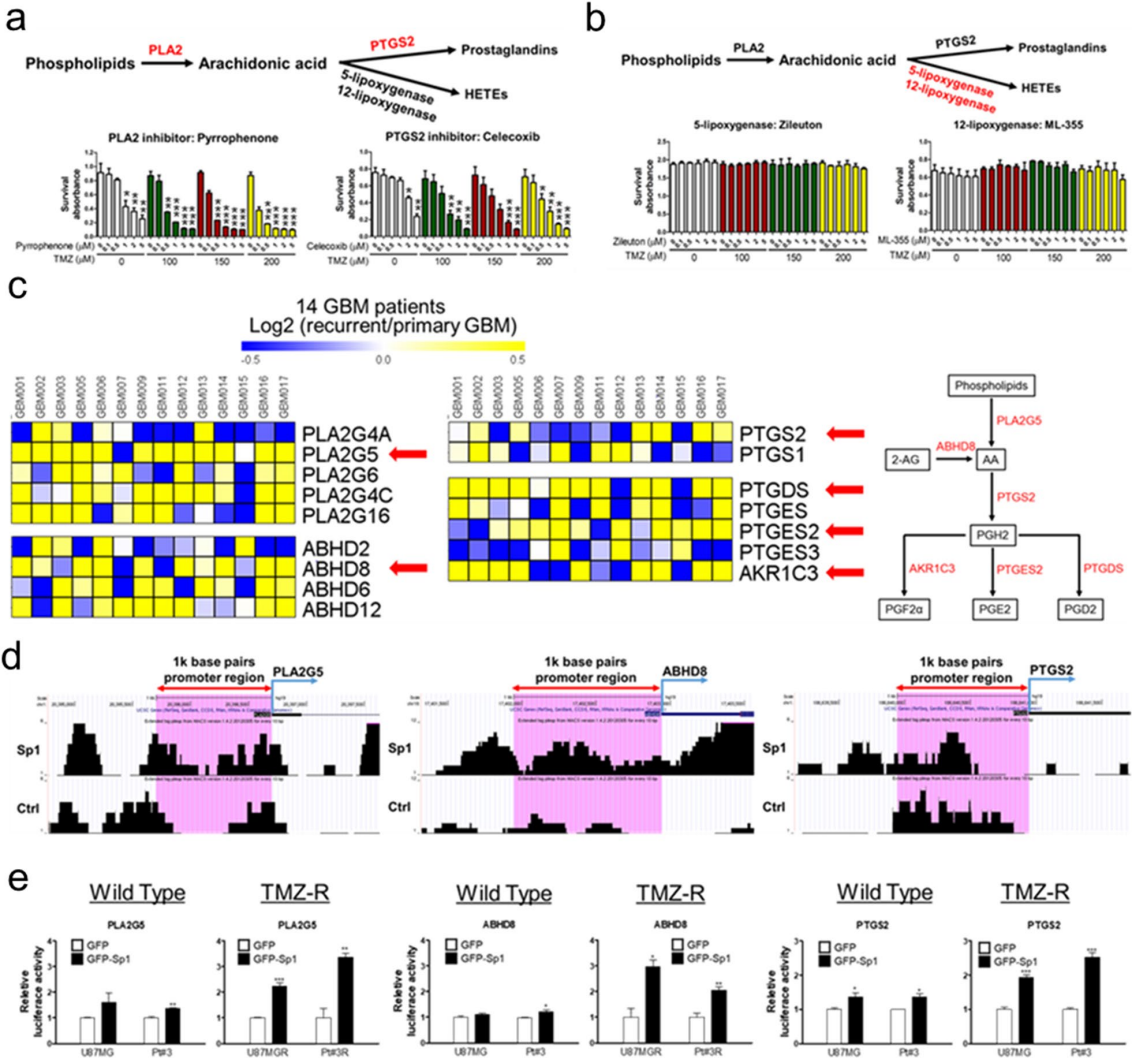
The Sp1-regulated COX2/PTGS pathway in recurrent glioblastoma. Effects of multiple inhibitors, including **a** Pyrrophenone (*left*) and celecoxib (*right*), **b** Zileuton (*left*) and ML-355 (*right*) on the viability of U87MG-R cells. The cells were treated with TMZ in the presence of the indicated inhibitors for 72 h, and cell viability was determined via MTT assay. **c** Paired primary and recurrent glioblastoma specimens. Samples were collected from 14 patients and subjected to RNA-Seq. The red arrow-marked genes play important roles in the metabolic pathway involving the synthesis of prostaglandins from AA (the COX pathway) as shown on the *right* panel. **d** Defined promoter regions of PLA2G5, ABHD8, and PTGS2. The binding regions of Sp1 were determined via ChIP-Seq. **e** Sp1-induced activities of pGL2-conjugated promoter constructs, including PLA2G5, ABHD8, and PTGS2. Luciferase reporter assay was employed to analyse the promoter activities in wild type and TMZ-resistant glioblastoma cells. Data were analysed by performing two-tailed unpaired Student’s *t* test

To elucidate the role of AA metabolism in TMZ-resistance acquisition, the effects of inhibitors that target PLA2, PTGS2/COX2, ALOX5, and ALOX12 on TMZ-resistant U87MG-R cells were evaluated. As shown in Fig. 2a, pyrrophenone (PLA2 inhibitor) and celecoxib (PTGS2 inhibitor) synergistically enhanced TMZ-induced cell death, whereas neither zileuton (ALOX5 inhibitor) nor ML-355 (ALOX12 inhibitor) exhibited any therapeutic effects (Fig. 2b). These results support the idea that the regulation of AA metabolism by Sp1, leading to the synthesis of prostaglandin and not lipoxygenase, is critical for the acquisition of TMZ resistance in GBM.

Moreover, to investigate the clinical relevance of Sp1-regulated prostaglandin synthesis, we collected 14 pairs of GBM specimens (primary vs. recurrent) for analysis (Additional file 1: Table S1). RNA-Seq revealed that the enzymes related to AA generation and prostaglandin metabolism, including PLA2G5, ABHD8, PTGS2, PTGES2, PTGDS, and AKR1C3, were obviously upregulated in the recurrent GBM specimens (Fig. 2c). Similar upregulated patterns were also identified in TMZ-resistant U87MG-R and Pt#3-R as compared with wild-type U87MG and Pt#3 cells, respectively (Additional file 1: Fig. S1). Additionally, chromatin immunoprecipitation (ChIP)-Seq and reporter assay analyses revealed that Sp1 significantly enhanced the transcriptional activities of PLA2G5, ABHD8, and PTGS2, with respect to prostaglandin synthesis in TMZ-resistant GBM cells by binding to their promoter regions (Fig. 2d, e). Overall, our results indicated the Sp1-regulated metabolic pathway from AA to prostaglandins is required for TMZ resistance acquisition in GBM.

### Enhancement of PGE2 synthesis in recurrent/resistant GBM

**Fig. 3:**
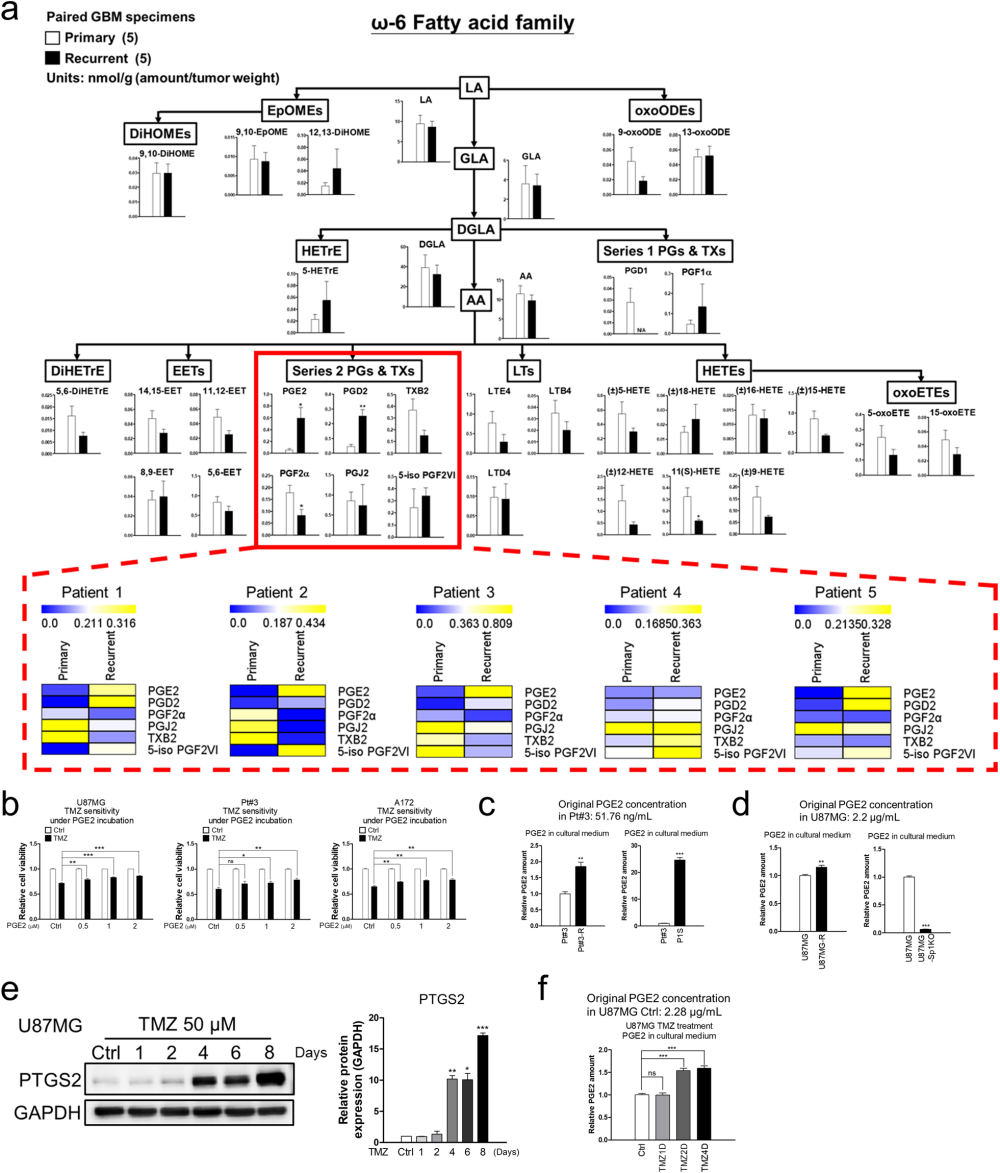
Effect of Sp1-regulated PGE2 production and secretion on TMZ-resistant glioblastoma. **a** Targeted ω-6 fatty acid metabolome analysis for paired primary and recurrent glioblastoma specimens from five patients based on UHPLC-QTOF-MS. Data in bar plots were analysed by performing two-tailed unpaired Student’s *t* test. The alteration in prostanoids from each patient was expressed as a heatmap. The full definitions of the different abbreviations are provided in Additional file 1: Table S6. **b** Effect of PGE2 on glioblastoma cell viability. After treatment with TMZ in the presence of PGE2 for four days, cell viability was estimated via MTT assay. **c, d** Level of PGE2 in culture media estimated via ELISA. **e, f** Cell lysates after treatment with TMZ for the indicated time interval analysed via Western blotting. The level of PGE2 in the culture media was determined via ELISA. Data and the quantifications were analysed by performing two-tailed unpaired Student’s t test

Further, targeted metabolome analysis focusing on ω-3/6 fatty acid metabolism was performed to determine the differences in the amounts of AA-related metabolites between paired (primary vs. recurrent) tissues from patients with GBM (Additional file 1: Table S2). As shown in Fig. 3a and Additional file 1: Fig. S2a, four out of a total five recurrent specimens exhibited significantly higher PGE2 and PGD2 levels. Additionally, we also estimated the amounts of AA-related metabolites via targeted arachidonate metabolome analysis. Thus, it was observed that the levels of PGD2 and PGE2 were also obviously higher in the recurrent tissue specimens than in normal brain tissue and primary specimens, suggesting that these two prostanoids are involved in TMZ resistance and tumour recurrence (Additional file 1: Fig. S2b). Further, as shown in Fig. 3b and Additional file 1: Fig. S2c, PGE2 significantly attenuated TMZ-induced cytotoxicity in patient-derived GBM cells, Pt#3, and U87MG. However, PGD2 did not affect cellular response to TMZ.

Besides, compared with the wild type glioblastoma cell line, an increase in PGE2 secretion was observed in TMZ-resistant glioblastoma cell lines (Pt#3-R, P1S, and U87MG-R cells), while a decrease was observed in U87MG-Sp1 cKO cells (Fig. 3c, d). Specifically, P1S cells, which exhibit TMZ resistance, secreted more PGE2 than patient-derived glioblastoma cells Pt#3 (Fig. 3c, right). Subsequently, the role of the PTGS2/PTGES2-mediated PGE2 synthesis in TMZ resistance was investigated. PTGS2 knockdown led to the enhancement of TMZ efficacy (Additional file 1: Fig. S3a) while PTGES2 overexpression attenuated the sensitivity of U87MG to TMZ (Additional file 1: Fig. S3b). Further, long term TMZ treatment resulted in the enhancement of PTGS2 expression and PGE2 secretion (Fig. 3e, f and Additional file 1: Fig. S4a-b). It was also observed that GBM cells, Pt#3 and U87MG, gradually showed increased tolerance to TMZ in the long-term treatment, and this increased tolerance was compromised by the PTGS2 inhibitor, celecoxib, resulting in a decrease in the number of adhered cells, an increase in the number of round-up cells, and the destruction of cell morphology (Additional file 1: Fig. S4c, d). These results supported the idea that the synthesis and secretion of PGE2 are significantly increased and required for the establishment of the recurrent and TMZ resistant GBM.

### Induction of mitochondrial fusion to rescue of TMZ-impaired respiration by PGE2 via EP1/EP3 cascades

**Fig. 4:**
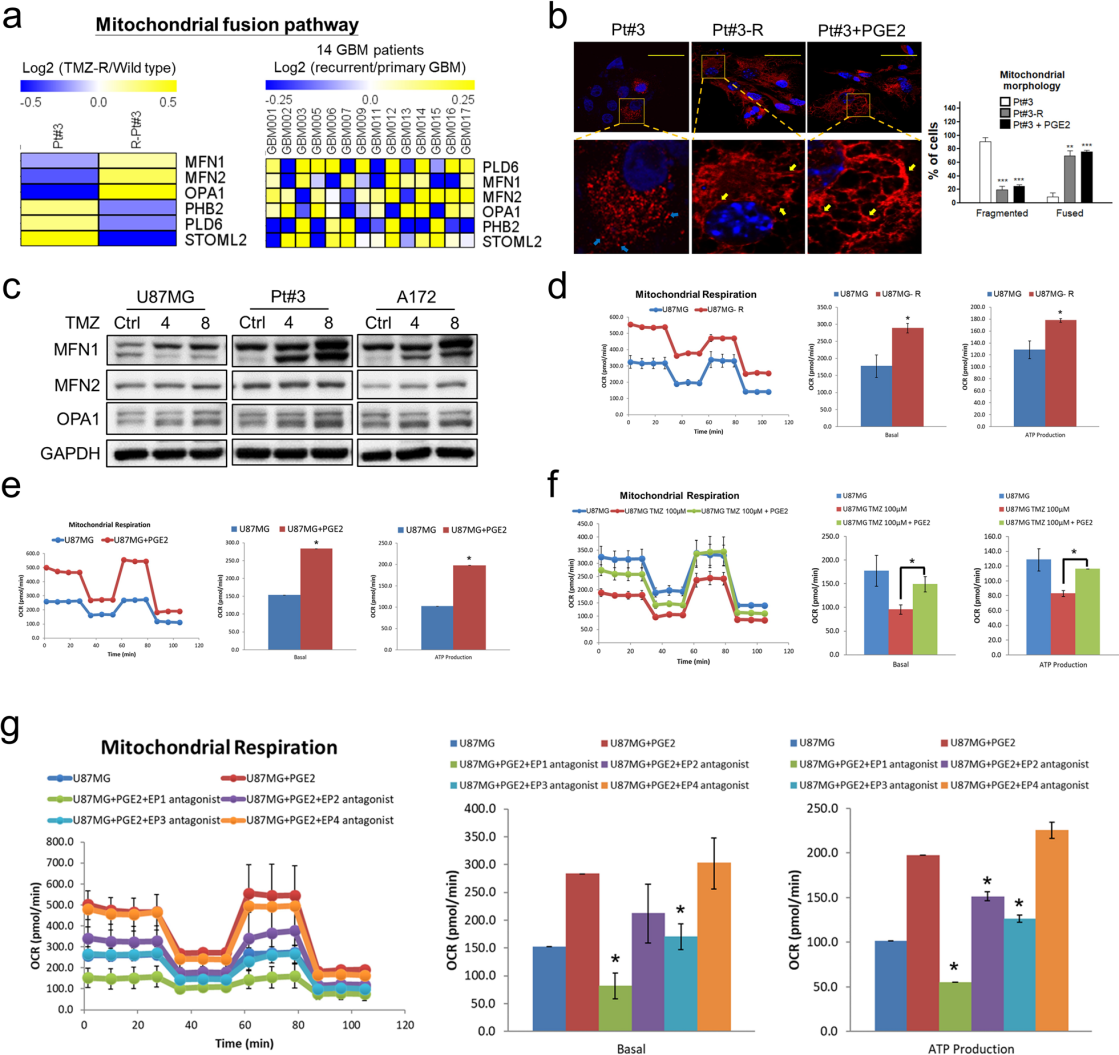
Effect of PGE2 on mitochondrial function. **a** RNA expression in cell line from patient with glioblastoma and paired glioblastoma specimens. **b** Mitochondrial morphology was fluorescently labelled by the pDsRed 2-Mito expression clone. Statistical data were analysed by performing two-tailed unpaired Student’s t test. Scale bar is 50 μm. **c** Effect of TMZ on mitochondrial fusion-related protein expression. **d** Mitochondrial activities of U87MG, U87MG-R, and U87MG-Sp1KO cells determined by performing Seahorse XF Mito Stress tests. **e** Mitochondrial activity determined via Seahorse XF Mito Stress tests after treatment with PGE2 for four days. **f** Mitochondrial activity after treatment with TMZ in the presence or absence of PGE2 for four days. **g** Mitochondrial activity after treatment with PGE2 in the presence of each antagonist for four days. Statistical data were analysed by performing two-tailed unpaired Student’s t test

Given that mitochondrial dynamics act as regulators in cancer processes [31–33], and that mitochondria play a critical role in the generation of bioenergy for GBM [18, 19], we evaluated whether mitochondrial fission and fusion are involved in drug resistance in GBM. Thus, RNA-Seq showed that mitochondrial fusion-related proteins, such as MFN1, MFN2, and OPA1, were significantly upregulated in resistant Pt#3-R and recurrent GBM specimens (Fig. 4a), while mitochondrial fission-related proteins showed a different pattern (Additional file 1: Fig. S5a). Further, we employed pDsRed2-Mito expression plasmid to fluorescently label mitochondrial in wild-type, TMZ-resistant, and PGE2-treated GBM cells. Unlike most of the mitochondrial was short and fragmented (fission) in wild-type GBM cells, mitochondria in TMZ-resistant and PGE2-treated GBM cells exhibited a network-like morphology (fusion) (Fig. 4b and Additional file 1: Fig. S5b), indicating that mitochondrial fusion is preferred in TMZ-resistant and PGE2-treated GBM cells. Moreover, consistent with PGE2 upregulation in long term TMZ-treated GBM cells, MFN1 and OPA1 levels showed a gradual increase (Fig. 4c). Taken together, results suggest that mitochondrial fusion, which enhances ATP production and respiration [31–33], is upregulated in TMZ-resistant GBM cells, and that PGE2 plays an important role in triggering mitochondrial fusion in TMZ-resistant GBM.

Further, Seahorse Mitochondrial Respiration Mito Stress Test and intracellular ATP measurement were used to evaluate the mitochondrial and total ATP production. The results showed an obviously enhanced mitochondrial activity in TMZ-resistant U87MG-R and Pt#3-R cells (Fig. 4d and Additional file 1: Fig. S5c), and most importantly, PGE2 also showed the ability to enhance ATP synthesis and mitochondrial activity in U87MG and Pt#3 cells (Fig. 4e and Additional file 1: Fig. S5c). Our results also revealed that PGE2 significantly attenuated TMZ-impaired mitochondrial respiration and rescues TMZ-impaired ATP synthesis (Fig. 4f and Additional file 1: Fig. S5e-f). Besides, Sp1 knockout suppressed mitochondrial respiration, while PGE2 significantly rescued the diminished mitochondrial activity and ATP production (Additional file 1: Fig. S5g-h). To identify the receptor responsible for PGE2-regulated mitochondrial activity, the effects of EP1–EP4 receptor antagonists (ONO-8713: EP1, PF-04418948: EP2, ONO-AE-240: EP3, and ONO-AE3-208: EP4) were investigated. Thus, it was observed that ONO-8713 and ONO-AE3-240, which inhibit EP1 and EP3 receptors, respectively, effectively attenuated the enhancement of ATP production by PGE2 (Fig. 4g and Additional file 1: Fig. S5i). Therefore, our results indicated that to establish TMZ resistance in GBM, Sp1-regulated PGE2 synthesis promoted mitochondrial fusion so as to enhance ATP production and rescue TMZ-impaired mitochondrial respiration through EP1 and EP3 cascades.

### PGE2-based promotion of FAO and TCA cycle progression contribute to increased ATP production

**Fig. 5:**
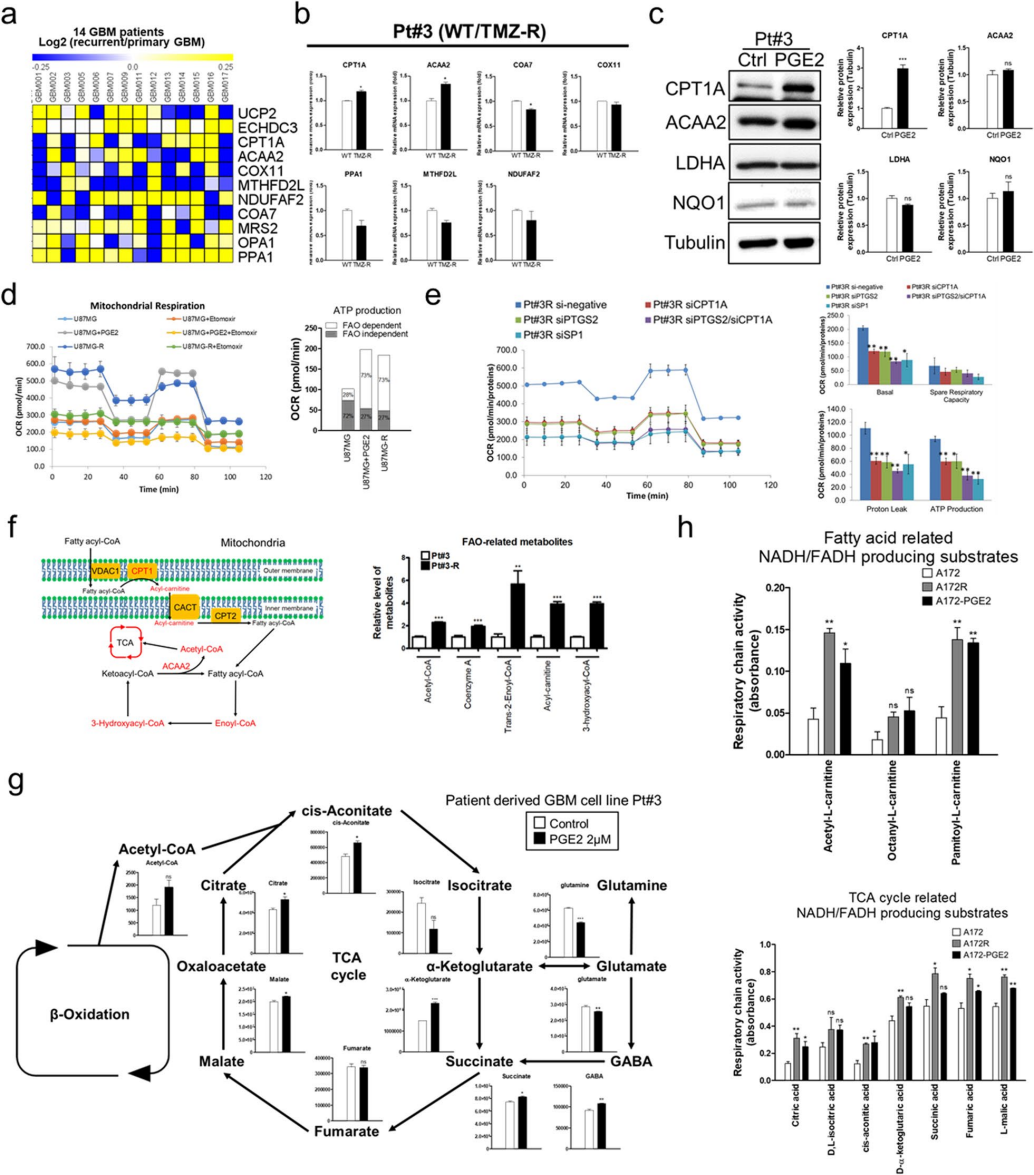
Effect of Sp1-regulated PGE2 on FAO and TCA cycles. **a** RNA expression in paired glioblastoma specimens. **b** Expression of mRNA in Pt#3 and Pt#3-R cells determined via real-time PCR. **c** Cell lysates after treatment with PGE2 for four days based on Western blotting. **d** Percentage of FAO measured by performing Seahorse XF Mito Stress Tests. **e** After transfection with Sp1, PTGS2, or CPT1A siRNA for three days, mitochondrial activity of the TMZ-resistant U87MG-R and Pt#3-R was determined. **f** *left* panel: FAO metabolic pathway; *right* panel: wild type Pt#3 and TMZ-resistant Pt#3-R cells subjected to metabolomics analysis. **g** Pt#3 cells after treatment with PGE2 for four days based on targeted metabolomics analysis. The alteration of the levels of the different metabolites is illustrated using bar plots. Data were analysed by performing two-tailed unpaired Student’s t test. **h** A172 and A172R cells after treatment with PGE2 for four days based on MitoPlate assay for 2 h. Data were analysed by performing two-tailed unpaired Student’s t test

In this study, we also investigated the contribution of the key proteins and metabolites that are regulated by Sp1 and PGE2 to the upregulation of ATP production after mitochondrial fusion [31–33]. In this regard, RNA-Seq revealed that Sp1 knockdown downregulated the expression of several mitochondrial ATP production-related genes, which were obviously upregulated in recurrent specimens (Table 1, Fig. 5a, and Additional file 1: Fig. S6a). Among these genes, CPT1A and ACAA2, which play predominant roles in the regulation of FAO pathways, were consistently and significantly upregulated in the recurrent GBM and TMZ-resistant GBM specimens (Fig. 5a-b, and Additional file 1: Fig. S6b). These results are consistent with our hypothesis that Sp1 enhances lipid metabolism in TMZ-resistant GBM, with CPT1A as the rate-limiting step of FAO [34], facilitating the transfer of fatty acids into the mitochondria for bioenergy generation. Therefore, we confirmed that PGE2 significantly enhances the protein expression of CPT1A without affecting the levels of glycolysis-related proteins, LDHA, and NQO1 (Fig. 5c, and Additional file 1: Fig. S4c). Moreover, PGE2 significantly increased the percentage contribution of FAO to mitochondrial respiration (Fig. 5d and Additional file 1: Fig. S6d). This is an important metabolic characteristic of TMZ-resistant GBM. Further, inhibiting Sp1, PTGS2, and CPT1A also decreased the mitochondrial respiration in TMZ-resistant GBM cells (Fig. 5e and Additional file 1: Fig. S6e). Knockdown of CPT1A sensitized TMZ-resistant cells to TMZ-suppressed cell proliferation (Additional file 1: Fig. S6h). Taken together, these results indicated that Sp1-regulated PGE2 enhances FAO by inducing CPT1A expression to acquire TMZ resistance.

Supporting the importance of FAO in drug resistance, untargeted metabolome analysis showed a significant increase in the levels of FAO-related metabolites, including acyl-carnitine, trans-2-Enoyl-CoA, 3-hydroxyacyl-CoA, and acetyl-CoA, in TMZ-resistant GBM cells (Fig. 5f). Consistent with this observation, for TCA cycle progression, PGE2 significantly increased the levels of citrate, cis-aconitate, α-ketoglutarate, succinate, and malate (Fig. 5g). Furthermore, to generate bioenergy, PGE2 enhanced the consumption of fatty acid- and TCA cycle-related substrates, such as short chain (Acetyl-L-carnitine) and long chain (Pamitoyl-L-carnitine) fatty acids, citric acid, cis-aconitic acid, fumaric acid, and L-malic acid, (Fig. 5h), implying that in the presence of PGE2, GBM cells show preference for the consumption of fatty acids as a major source of bioenergy. Therefore, Sp1-regulated PGE2 increases the number of metabolites in FAO and drives TCA cycle progression after mitochondrial fusion to rescue TMZ-induced nutrient deprivation and mitochondria damage.

### Blockade of PGE2 action by EP1 and EP3 antagonists suppresses TMZ-resistant GBM

**Fig. 6:**
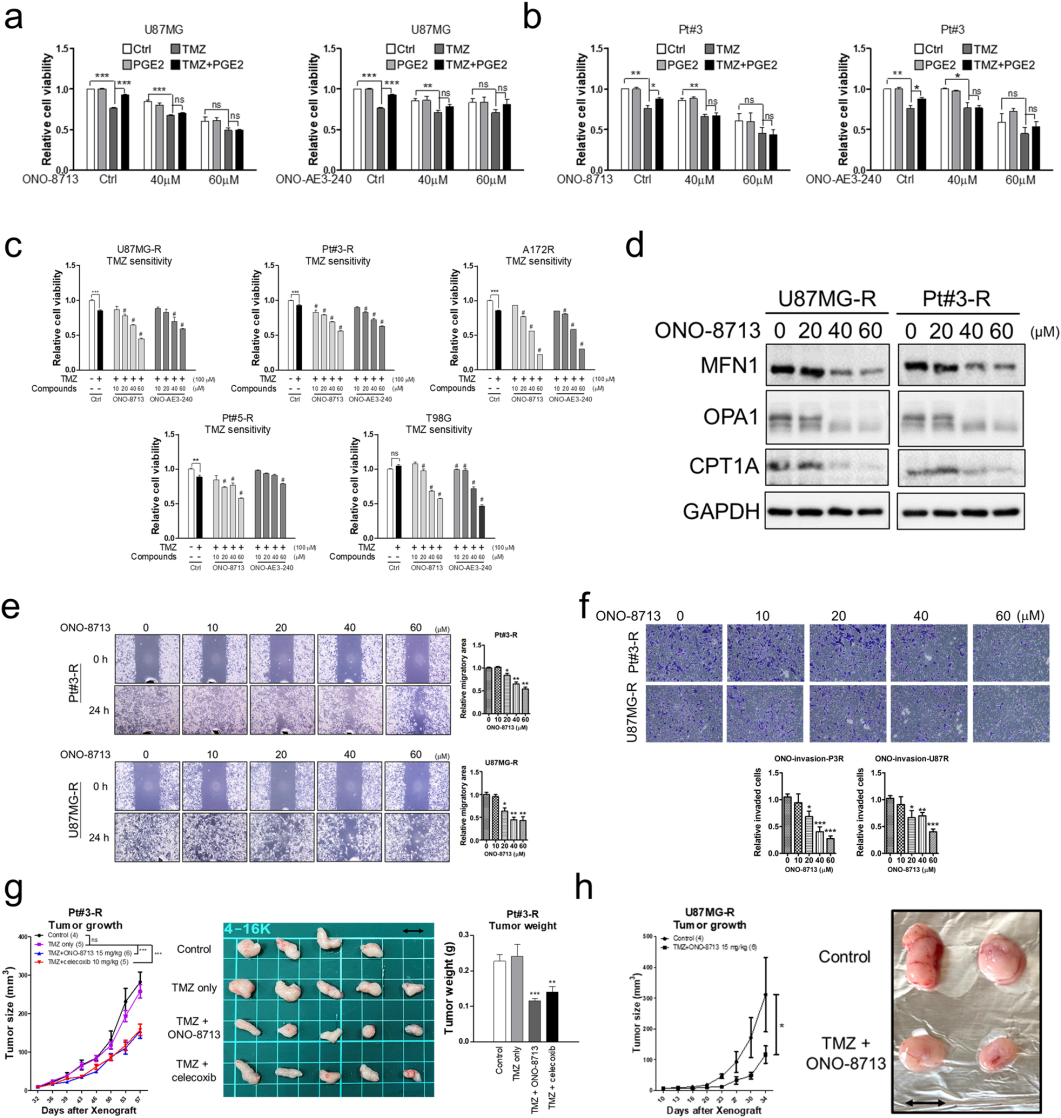
Therapeutic effect of EP antagonists on TMZ-resistant glioblastoma. **a**, **b** Effect of EP1 antagonist, ONO-8713, or EP3 antagonist, ONO-AE3-240, on glioblastoma cells. The cells were treated with ONO-8713 or ONO-AE3-240 in the presence of TMZ and PGE2 for 96 h. Cell viability was determined via MTT assay. **c** TMZ-resistant glioblastoma cells treated with different compounds in the presence of TMZ for 96 h. Cell viability was determined by performing MTT assay. (# implies a significant difference compared with the TMZ-treated group). **d** After treatment with ONO-8713 for four days, cell lysates were prepared for Western blotting. **e, f** After treatment for 48 h, migratory and invasive activities of TMZ-resistant GBM cells were determined by wound-healing and transwell invasion assays, respectively. **g, h** Effect of ONO-8713 or celecoxib on the growth of TMZ-resistant cell-derived tumour in xenograft mouse model. The difference between the control and experimental groups was analysed by performing two-way ANOVA. The scale bar is 1 cm

To prevent GBM from acquiring PGE2-based TMZ resistance, several compounds targeting PGE2 synthesis (celecoxib) and receptors (ONO-8713, PF-04418948, ONO-AE3-240, ONO-AE3-208) were evaluated. Among the candidate PGE2 receptor-targeting compounds, the EP1 antagonist, ONO-8713, and the EP3 antagonist, ONO-AE3-240, significantly abolished PGE2-induced resistance (Fig. 6a, b and Additional file 1: Fig. S7a, b). Additionally, ONO-8713 and ONO-AE3-240 significantly inhibited the survival of multiple GBM cells with or without TMZ resistance, including T98G cells expressing MGMT (Fig. 6c and Additional file 1: Fig. S7c). These results suggest that, in addition to suppressing wild-type GBM cells, ONO-8713 significantly suppresses the survival of TMZ-resistant GBM cells. Further, we evaluated the mechanism of ONO-8713 in suppressing TMZ-resistant GBM cells. In Fig. 6d, ONO-8713 significantly inhibited mitochondrial fusion and FAO characterized by MFN1/OPA1 and CPT1A expression, respectively, in TMZ-resistant GBM cells. The results suggested that ONO-8713 blocks the PGE2-induced mitochondrial fusion and FAO, which is involved in cancer cell migration and invasion [20, 21]. Hence, we showed that blocking PGE2 function by ONO-8713 and celecoxib significantly suppressed TMZ-resistant GBM migration and invasion (Fig. 6e, f and Additional file 1: Fig. S8).

As TMZ is known to induce DNA damage, we attempted to determine whether PGE2-induced TMZ resistance is mediated through modulating DNA damage response. Results showed that PGE2 was unable to reverse the DNA damage induced by TMZ in GBM cells characterized by pRad50, p-Chk2 and γH2Ax levels (Additional file 1: Fig. S9a). Moreover, ONO-8713 targeting the PGE2 receptor, Etomoxir targeting CPT1A, and celecoxib targeting PGE2 synthesis did not enhance TMZ-induced DNA damage (Additional file 1: Fig S9b-d). These results suggest that PGE2-induced mitochondrial activity is a novel mechanism triggering TMZ resistance in GBM. Based on the determined therapeutic potential in vitro, we evaluated the therapeutic effect of ONO-8713 in suppressing resistant GBM tumour growth in the mouse xenograft tumour model. As shown in Fig. 6g, both ONO-8713 and celecoxib significantly inhibited Pt#3-R tumour growth. Additionally, ONO-8713 also significantly inhibited U87MG-R-derived tumour growth (Fig. 6h).

**Fig. 7:**
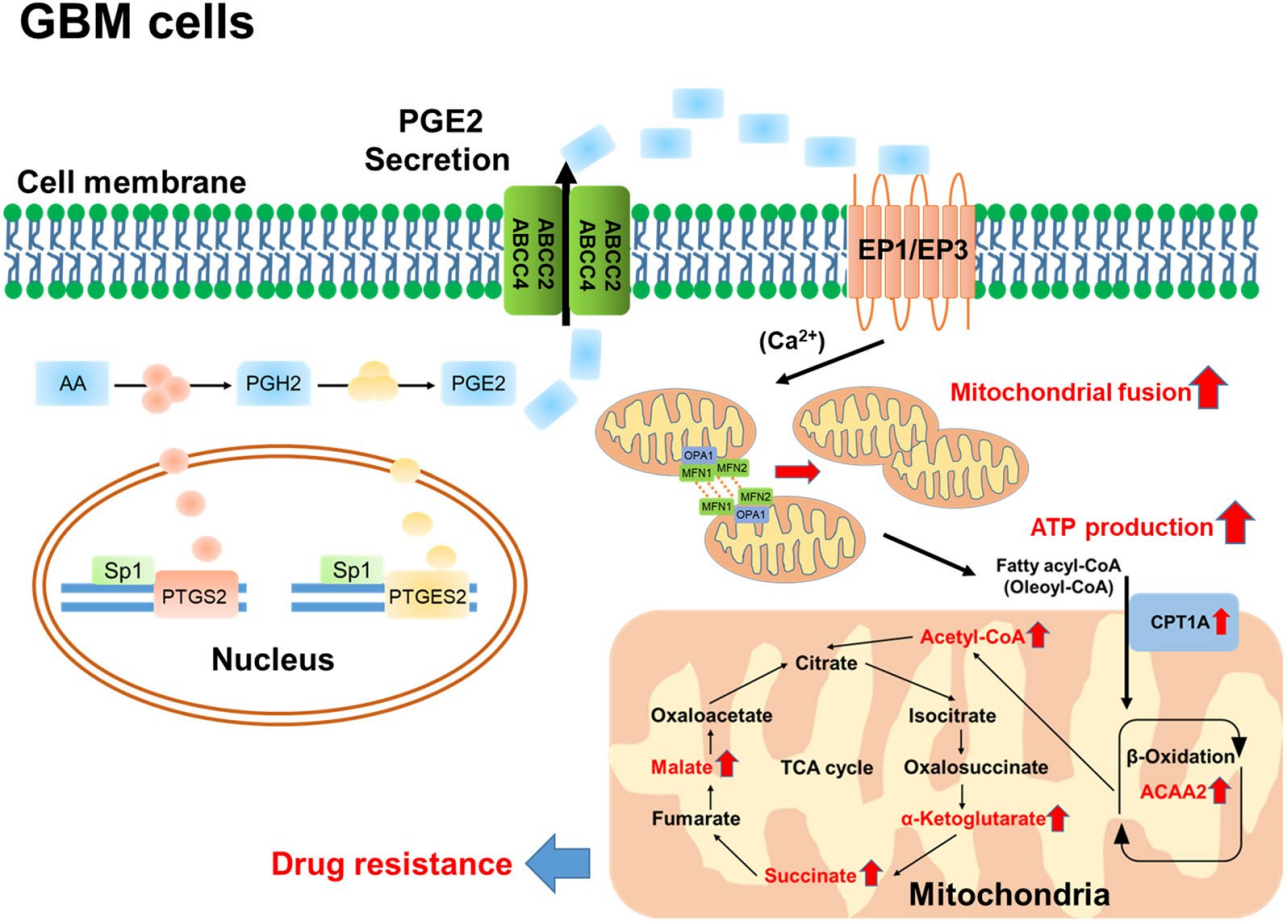
Schematic representation of the working model. According to established gene expression profiles and ω-3/6 fatty acid metabolome, we found that Sp1 increased the PTGS2 expression and PGE2 production in recurrent GBM patients and in TMZ-resistant GBM cells. For inducing TMZ resistance, PGE2 activated mitochondrial FAO and TCA cycle progression through enhancing mitochondrial fusion. These results showing the role of PGE2 metabolism in GBM provide us a new strategy to attenuate drug resistance or to re-sensitize recurred glioblastoma

Taken together, through enhancing the FAO and TCA cycle, Sp1-regulated PGE2 upregulation increases mitochondrial ATP production, which sustains cell survival, leading to the increase in the tolerance of GBM cells in response to TMZ-mediated chemotherapy (Fig. 7). Importantly, in TMZ-resistant GBM cells exhibiting upregulated oxygen consumption, ONO-8713 is able to block PGE2-induced mitochondrial function, resulting in energy exhaustion followed by sensitization of GBM to TMZ treatment. Therefore, TMZ treatment combined with EP1 receptor antagonist is a potential therapeutic application for GBM patients.

## Discussion

Given that GBM utilizes mitochondrial oxidation to generate bioenergy [18, 19], we investigated which part of the ATP-generating system is upregulated in TMZ-resistant GBM. Our study indicated that FAO and TCA cycles are upregulated in TMZ-resistant GBM owing to the action of PGE2. It has also been reported that FAO plays an important role in tumour development in nutrient-deprived environment [19] and cancer metastasis [20, 21]. Particularly, increases fatty acid uptake and the alteration of lipid metabolism are associated with drug resistance in cancers and provide the essential bioenergy for cancer survival based on the enhancement of FAO efficacy [35]. For example, the resistance of cancers to antiangiogenic drugs is due to the alteration of FAO and lipid metabolism in the tumour environment [36]. Therefore, therapeutic strategies that target the alteration of lipid metabolism, such as blocking cancer-associated adipocyte-mediated fatty acid production followed by the inhibition of free fatty acid uptake by cancer cells, have been designed to combat drug resistance [36]. However, strategies that target FAO for the treatment of drug resistant cancer remain limited. Even though etomoxir reportedly blocks FAO by inhibiting CPT1 and suppressing GBM cell growth [19], our experimental results revealed that etomoxir is not effective in inhibiting drug resistance in GBM (data not shown). Hence, the PGE2/EP1 cascade, which has a significant regulatory effect on CPT1-dependent FAO, is expected to be a potential target for overcoming drug resistance.

It has been shown that mitochondrial dysregulation participates in cancer drug resistance. Mitochondria retrograde signaling pathways, the communication between the dysfunctional mitochondria and the nuclear genetic compartment, modulate the gene expression leading to drug resistance [37]. Moreover, mitochondria transfer between different cell types in the tumor environment [38] is associated with chemo-resistance. Mitochondria dynamics, including fusion and fission, are reported to be involved in cancer progression, balancing bioenergy and ROS production, and maintaining the metabolic homeostasis [32]. However, the role of mitochondrial dynamics in drug resistance is still controversial. Our study suggested that TMZ-resistant GBM enhances mitochondrial fusion, which results in the elevation of oxygen consumption and bioenergy production, indicating that mitochondrial activation is required for acquiring resistance. Moreover, although it has been reported that TMZ induces the damage of mitochondrial function [39] and that TMZ-resistant gliomas exhibit higher mitochondrial respiration [40], the mechanism of TMZ-induced mitochondrial reprogramming to establish drug resistance is still unclear. Herein, we showed that U87MG-R, as compared with U87MG, exhibited higher basal respiratory activity, whereas PGE2 significantly increased overall respiratory activity, especially maximal respiratory capacity. The discrepancy between U87MG-R vs. U87MG and U87MG-PGE2 vs. U87MG might be caused by the different process of acquiring TMZ resistance. TMZ-resistant cells were generated from cells treated with TMZ for at least 3 months. TMZ-resistant cells keep basal mitochondrial activity at highly working level to adapt TMZ-induced energy deprivation. Hence, FCCP-induced maximal respiratory capacity in TMZ-resistant cells was not obvious as compared with wild-type cells. In contrast, PGE2 treatment was given for only four days. It is more likely to be a transient upregulation of mitochondria-driven ATP synthesis induced by FCCP. Moreover, while we knockdown PTGS2, the maximal respiratory capacity was decreased. The results suggested that PGE2 plays an important role in promoting maximum respiratory capacity of mitochondrial in TMZ-resistant GBM cells.

Celecoxib is a well-known COX2/PTGS2 inhibitor that attenuates the synthesis of PGE2, and reportedly, kills GBM cells [41]. However, it failed a phase II clinical trial owing to its poor permeability with respect to crossing the blood–brain barrier [42, 43]. Therefore, a treatment strategy that targets the PGE2-induced functional pathway, such as PGE2 receptor (EP1-EP4) blocking, instead of COX2/PTGS2-mediated PGE2 synthesis suppression, is an alternative medical option. Reportedly, PGE2 receptors are associated with cancer malignancy, while their antagonists have also shown tumorigenesis inhibition effects [44]. Among EP1–EP4 receptors, EP1 is involved in the activation of cancer cell migration and invasion [45–47], supports tumour adaptation to hypoxia [48], and enhances cancer initiation [49, 50]. EP2 is known to be involved in the induction of angiogenesis [44] and in the suppression of antitumor immune response [51]. Further, its inhibition in glioma results in tumour growth suppression and cell migration/ invasion inhibition [52]. Conversely, the role of EP3 in tumorigenesis is still controversial [44]. The results of our previous study showed that the EP3 antagonist, ONO-AE3-240, also exerts cell cytotoxicity on TMZ-resistant GBM cells. Additionally, it has been demonstrated that EP4 is involved in cancer migration, metastasis, and aberrant DNA methylation [53–55]. The results of this study indicated that the EP1 antagonist, ONO-8713, is capable of blocking PGE2-induced mitochondrial activation and suppressing the survival of TMZ-resistant GBM cells. Therefore, EP antagonists, instead of celecoxib, have potential to attenuate drug resistance by blocking PGE2-mediated signalling in GBM. Overall, PGE2 induces TMZ resistance in GBM via mitochondria-mediated FAO activation under the control of EP1 and EP3. Further combining TMZ with an EP1 antagonist present as a potential combination therapeutic strategy for TMZ-resistant GBM. Therefore, the development of EP1 antagonists that can cross the blood–brain barrier during the treatment of TMZ-resistant GBM have great application prospects.

## Conclusion

Sp1 increases PGE2 synthesis through enhancing gene expression involved in AA metabolism to PGE2 in recurrent GBM, leading to TMZ resistance. Further, PGE2 increases mitochondrial fusion, resulted in the enhancement of FAO and TCA cycle to increase the ATP production, through the EP1 and EP3 receptors. Moreover, EP1 antagonist ONO-8713 exhibits a potentially therapeutic effect on TMZ-resistant GBM in vivo and in vitro (Fig. 7).

## Supplementary Information

**Additional file 1**. Additional figures and tables.

## Data Availability

The author declared that all and the other data supporting the findings of this study are available within the paper. The raw data that support the findings of this study are available from the corresponding author upon reasonable request.

## Abbreviations

AA: Arachidonic acid
ABC: ATP binding cassette;
ABHD: Abhydrolase domain containing
ACAA: Acetyl-coenzyme A acyltransferase
AKR1C3: Aldo–keto reductase family 1 member C3
ALOX: Arachidonate lipoxygenase
cAMP: Cyclic adenosine 3',5'-cyclic monophosphates
COX2: Cyclooxygenase-2
ChIP: Chromatin immunoprecipitation
CPT: Carnitine palmitoyltransferase
CYP: Cytochrome p450
ENO2: Enolase 2
EP: E-type prostanoid receptor
FAO: Fatty acid β-oxidation
GBM: Glioblastoma
GFA: Glial fibrillary acidic protein
HETE: Hydroxyeicosatetraenoic
IDH-1: Isocitrate dehydrogenase 1
LDHA: Lactate dehydrogenase A
MFN1/2: Mitofusin ½
MGMT: O6-methylguanine-DNA methyltransferase
OPA1: Optic Atrophy 1
PDK1: Pyruvate dehydrogenase kinase-1
PG: Prostaglandin
PLA2G5: Phospholipase A2 group V
PTGES2: Prostaglandin E synthase 2
PTGS2: Prostaglandin-endoperoxide synthase 2
Sp1: Specificity protein 1
TCA: Tricarboxylic acid
TEM: Transmission electron microscopy
TMZ: Temozolomide
