# Supplementary material for: Reprogramming of arachidonate metabolism confers temozolomide resistance to glioblastoma through enhancing mitochondrial activity in fatty acid oxidation
Source: J Biomed Sci. 2022 Mar 25;29:21. doi: 10.1186/s12929-022-00804-3 (PMC8952270; doi:10.1186/s12929-022-00804-3)
Supplement: Supplementary file 1 — Additional file 1. Additional figures and tables. [file 12929_2022_804_MOESM1_ESM.docx]

**Supplementary information**

**Reprogramming of arachidonate metabolism confers temozolomide resistance to glioblastoma through enhancing mitochondrial activity in fatty acid oxidation**

Yu-Ting Tsai^1,2^**^†^**, Wei-Lun Lo^3,4,5^**^†^**, Pin-Yuan Chen^6,7,8^, Wen-Bing Yang^1,5^, Chiung-Yuan Ko^5,9,10,11^, Jian-Ying Chuang^5,9,10,11,12^, Tzu-Jen Kao^5,9,10,11^, Kwang-Yu Chang^12^, Chia-Yang Hung^13^, Ushio Kikkawa^1^, Wen-Chang Chang^1,5^*, Tsung-I Hsu^5,9,10,11,12^*

**^†^These authors contributed equally to this work.**

^1^ Graduate Institute of Medical Sciences, College of Medicine, Taipei Medical University, Taipei 110, Taiwan

^2^ Department of Cancer Biology, Wake Forest University School of Medicine, Winston-Salem, NC 27101, USA.

^3^ Department of Surgery, School of Medicine, College of Medicine, Taipei Medical University, Taipei 110, Taiwan

^4^ Department of Neurosurgery, Shuang Ho Hospital, Taipei Medical University, Taipei 110, Taiwan

^5^ TMU Research Center of Neuroscience, Taipei Medical University, Taipei, Taiwan 110

^6^ School of Medicine, Chang Gung University, Taoyuan City, Taiwan 33302

^7^ Department of Neurosurgery, Keelung Chang Gung Memorial Hospital, Keelung, Taiwan 204

^8^ Department of Neurosurgery, Linkou Chang Gung Memorial Hospital, Taoyuan, Taiwan 333

^9^ Graduate Institute of Neural Regenerative Medicine, College of Medical Science and Technology, Taipei Medical University, Taipei, Taiwan 110

^10^ Ph.D. Program for Neural Regenerative Medicine, College of Medical Science and Technology, Taipei Medical University and National Health Research Institutes, Taipei, Taiwan 110.

^10^ Ph.D. Program in Medical Neuroscience, College of Medical Science and Technology, Taipei Medical University and National Health Research Institutes, Taipei, Taiwan 110.

^11^ TMU Research Center of Cancer Translational Medicine, Taipei, Taiwan 110

^12^ National Institute of Cancer Research, National Health Research Institutes, Tainan, Taiwan 704

^13^ Department of Immuno-Oncology, Beckman Research Institute, City of Hope, Duarte, CA, USA 91010

Correspondence to: Tsung-I Hsu and Wen-Chang Chang

**Supplementary figures and figure legends**


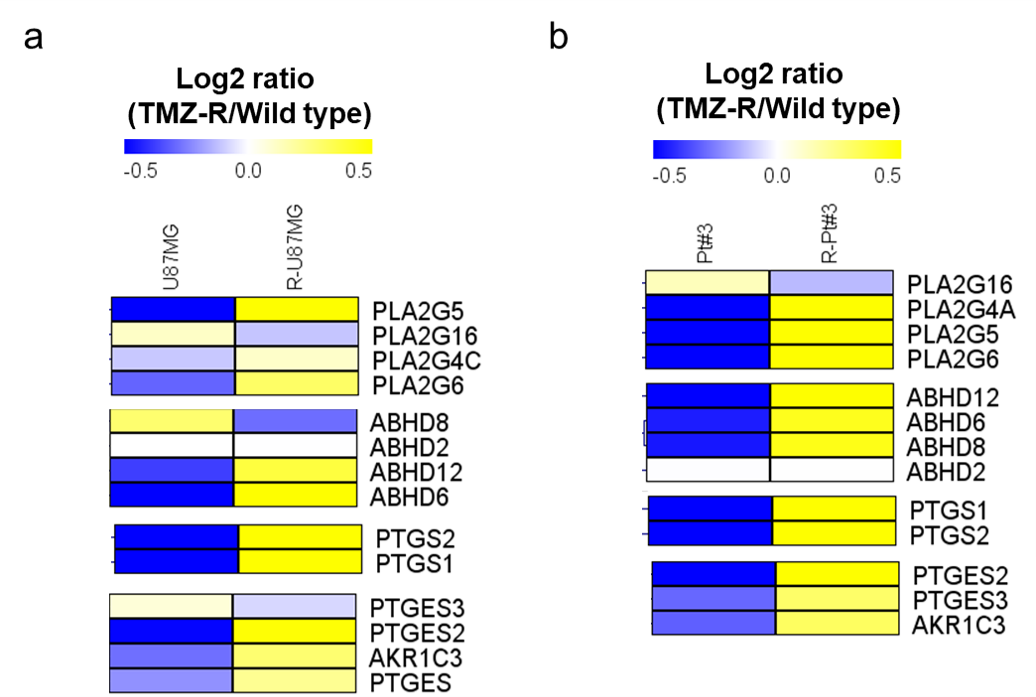


**Supplementary Figure S1. Gene expression of enzymes related to AA generation and prostaglandin metabolism.** RNA expression was analyzed by RNA-Seq in paired **(a)** GBM cell line and **(b)** GBM patient-derived cells.


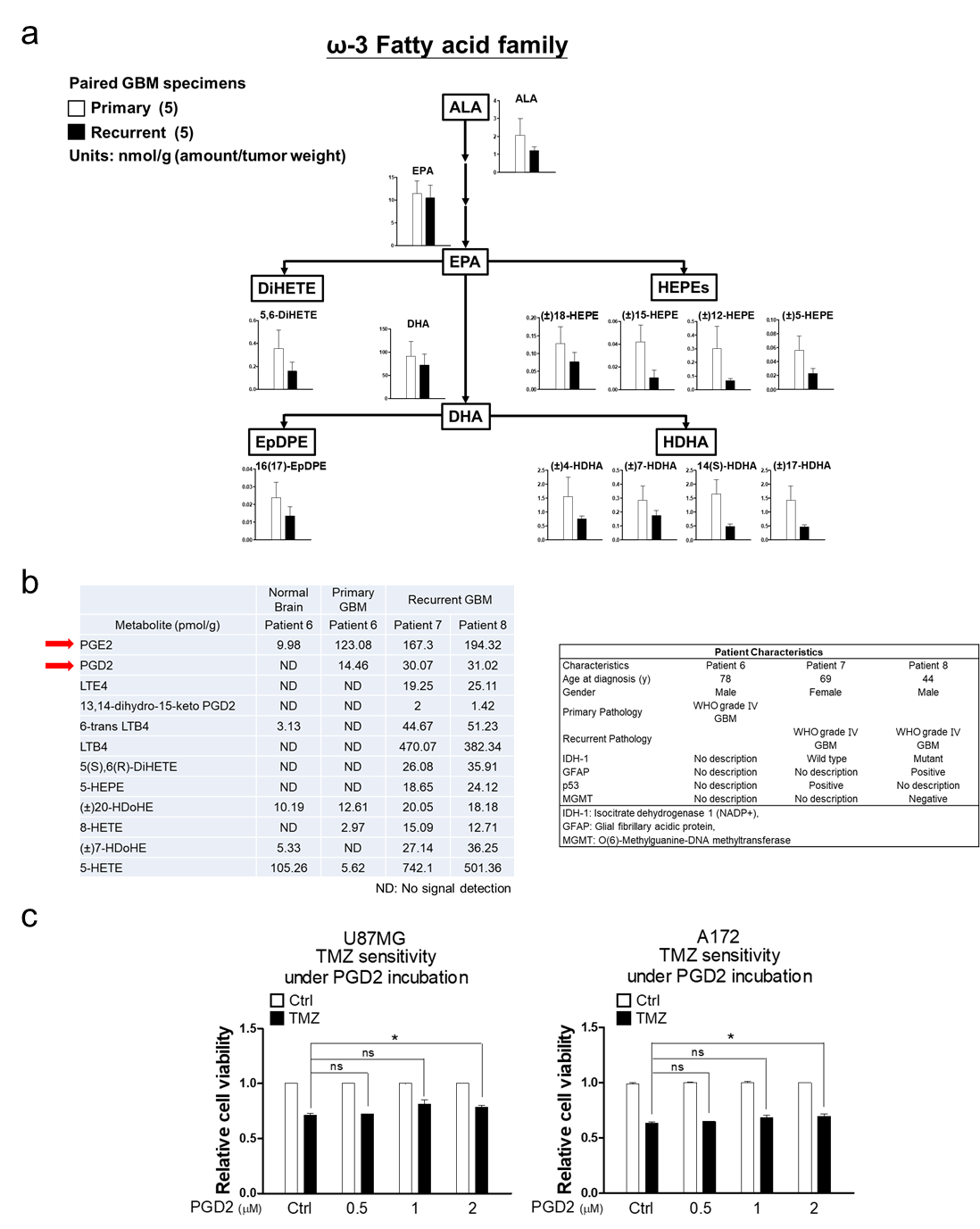


**Supplementary Figure S2. The amount of AA-related metabolites in glioblastoma tumors. (a)** Targeted ω-3 fatty acid metabolome for paired primary and recurrent glioblastoma specimens from 5 patients determined by UHPLC-QTOF-MS. Data in bar plots were analyzed by two-tailed unpaired Student’s t test. **(b)** Left pannel: Targeted arachidonate metabolome for normal brain tissue, primary and recurrent glioblastoma specimens determined by UHPLC-QTOF-MS. Right panel: Patients’ characteristics. Red arrows indicate two significantly increased metabolites in recurrent glioblastoma specimans. **(c)** After treatment with TMZ in the presence of PGD2 for 4 days, cell viability was estimated by MTT assay. Data were analyzed by two-tailed unpaired Student’s t test (n=3).


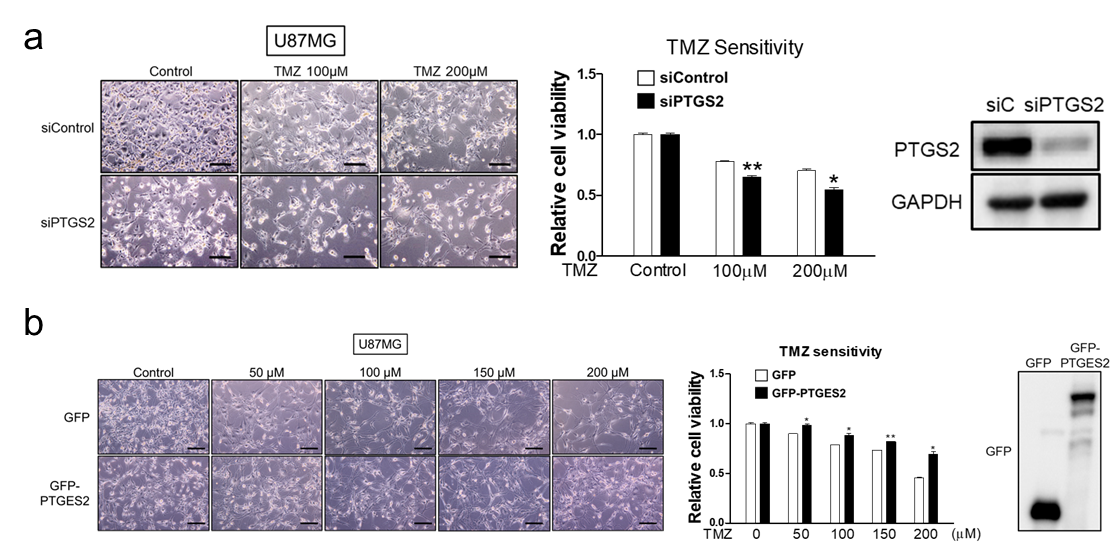


**Supplementary Figure S3. Effect of PGE2 production on cellular response to TMZ. (a)** Effect of PTGS2 knockdown or **(b)** PTGES2 overexpression on viability in response to TMZ treatment for four days. Data were analyzed by two-tailed unpaired Student’s t test (n=3). PTGS2 knockdown and GFP-PTGES2 overexpression were confirmed by Western blotting. The scale bar is 0.2 mm.


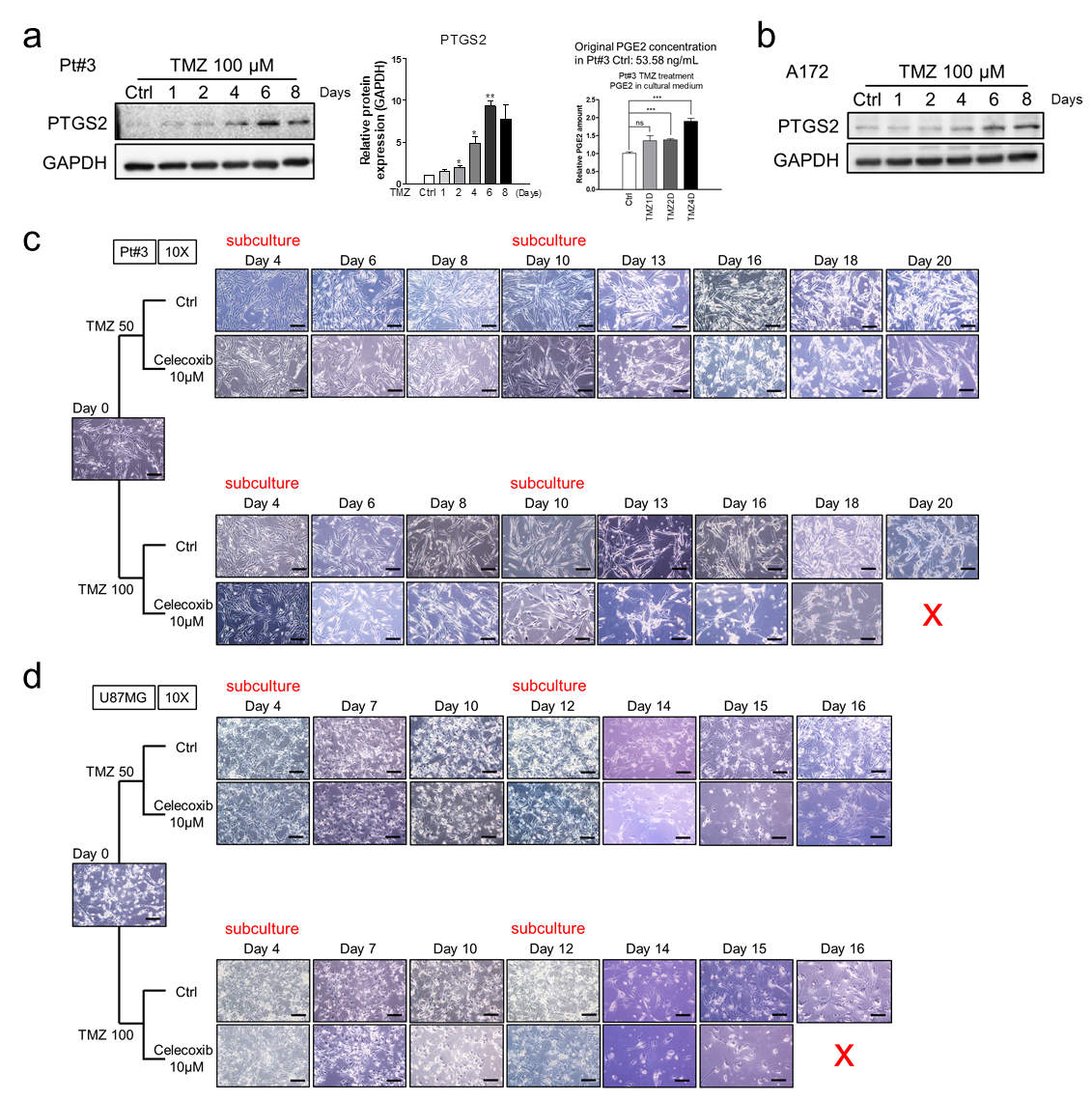


**Supplementary Figure S4. Effect of celecoxib on cellular response to TMZ. (a**, **b)** After treatment with TMZ for indicated time interval, cell lysates were collected for Western blotting using indicated antibodies. The level of PGE2 was confirmed by ELISA. Data with quantification were analyzed by two-tailed unpaired Student’s t test (n=2). **(c)**, **(d)** U87MG cells and Pt#3 were cultured in the presence of TMZ with or without celecoxib for indicated time period, and morphology was photographed under 10X magnification. “Red X” means no survival cells in the culture dishes. The scale bar is 0.2 mm.


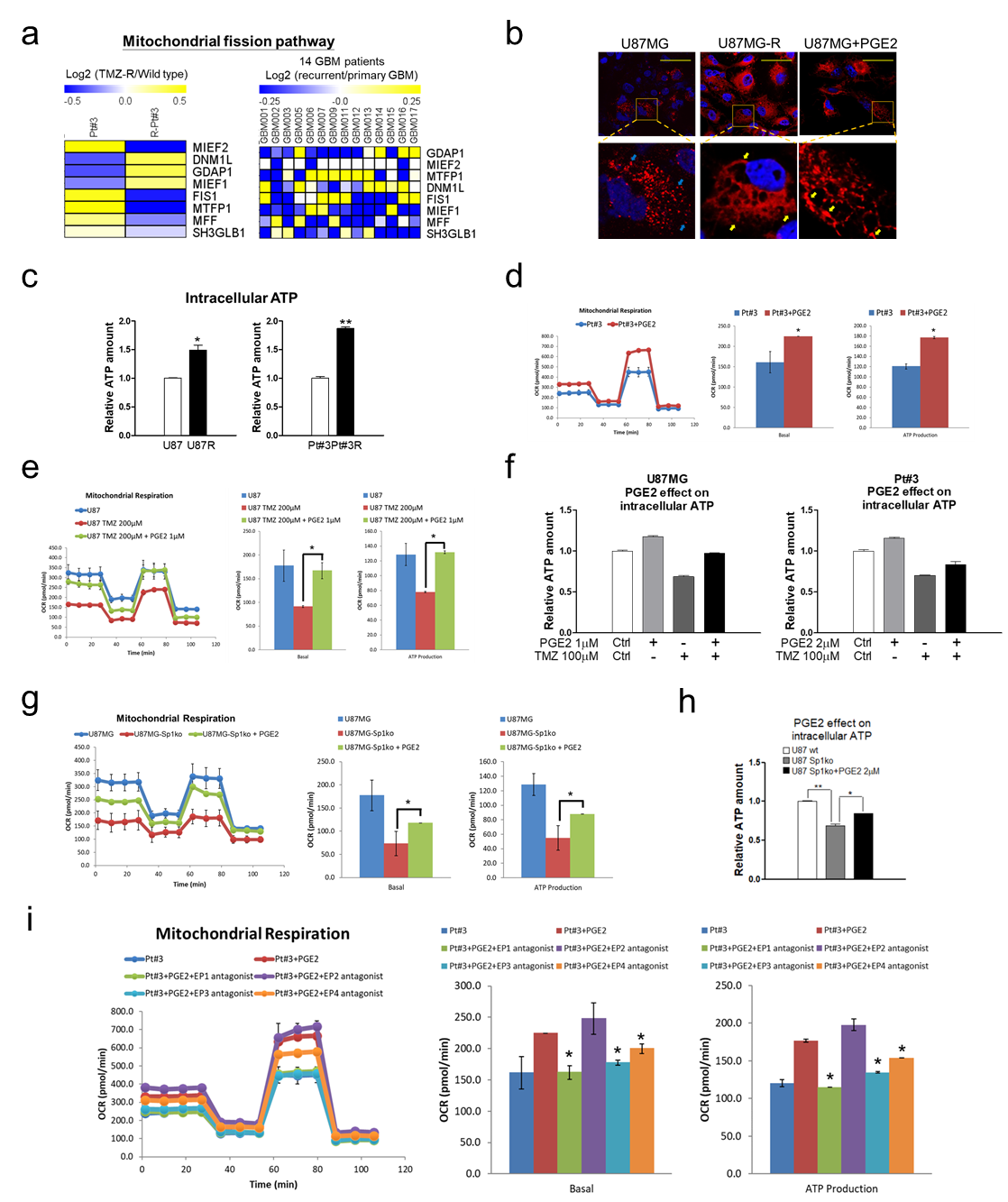


**Supplementary Figure S5. The effect of Sp1-regulated PGE2 on mitochondrial activity. (a)** RNA expression was analyzed in paired glioblastoma patient-derived cell line and glioblastoma specimens. **(b)** Mitochondrial morphology was determined by pDsRed 2-Mito vector-mediated IF staining. The scale bar is 50 μm. **(c)** The intracellular ATP mount measured by ATP Colorimetric/Fluorometric Assay. **(d)**, **(e)**, **(f), (g), (h)** After treatment with PGE2 for 4 days, mitochondrial activity was determined by the Seahorse XF Mito Stress test. And, the intracellular ATP mount measured by ATP Colorimetric / Fluorometric Assay. Data were analyzed by two-tailed unpaired Student’s t test. **(i)** After treatment with PGE2 in the presence of EP1-EP4 antagonists for 4 days, mitochondrial activity was determined by the Seahorse XF Mito Stress test. Data were analyzed by two-tailed unpaired Student’s t test.


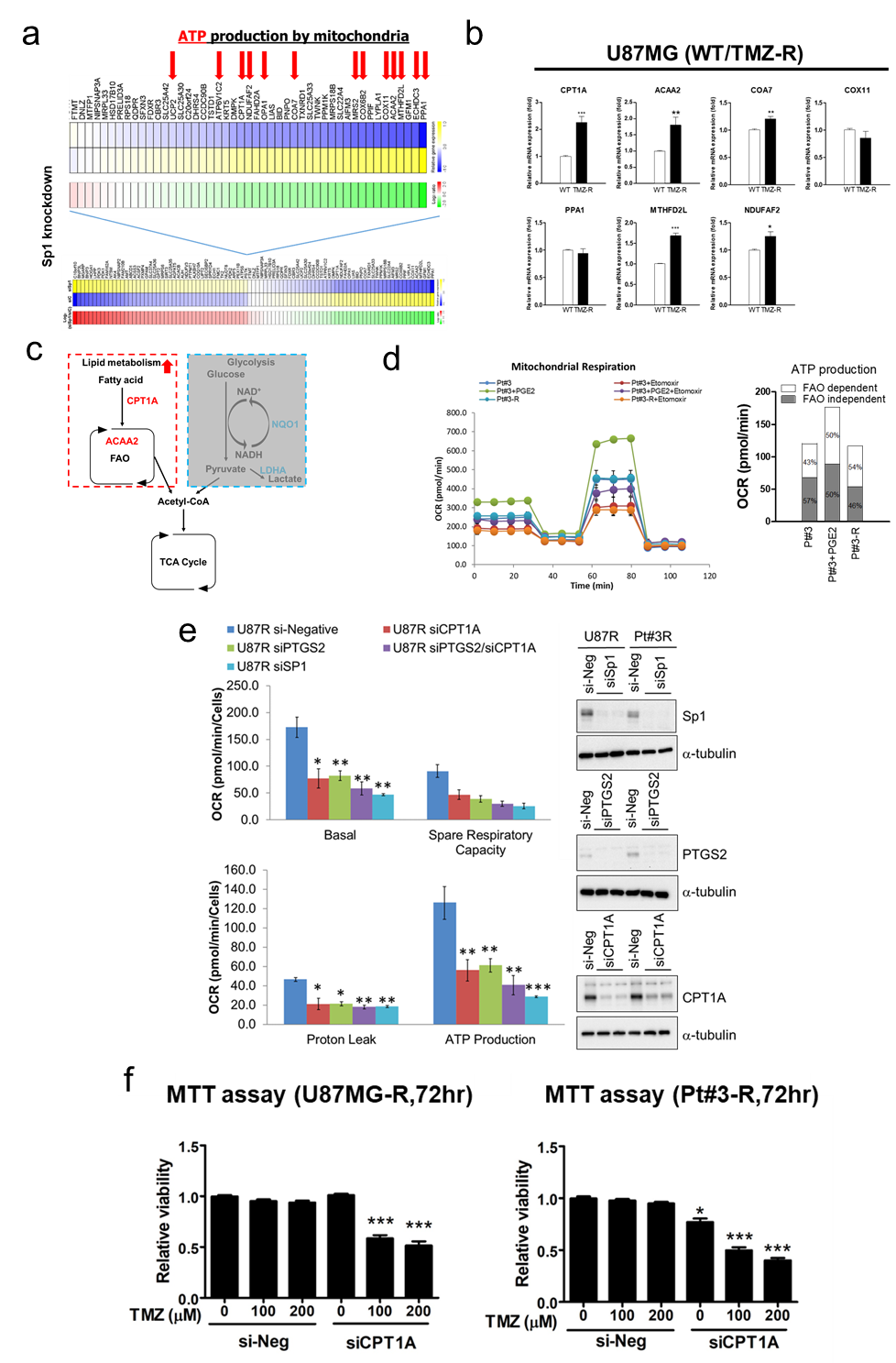


**Supplementary Figure S6. The effect of Sp1-regulated PGE2 on FAO. (a)** Effect of Sp1 knockdown in U87MG cells on mitochondria-related gene expression. Genes which are essential for ATP production are indicated. **(b)**The expression of mRNA was measured by real-time PCR. Data were analyzed by two-tailed unpaired Student’s t test (n=3). **(c)** FAO and glycolysis pathway. **(d)** The percentage of FAO was measured by the Seahorse XF Mito Stress Test. **(e)** After transfection with Sp1, PTGS2, or CPT1A siRNA for three days, mitochondrial activity in the TMZ-resistant GBM cells was determined. Effect of Sp1, PTGS2, and CPT1A knockdown were confirmed by Western blotting. **(f)** Effect of CPT1A knockdown on TMZ-resistant GBM cell viability in response to TMZ treatment for three days. Data were analyzed by two-tailed unpaired Student’s t test.


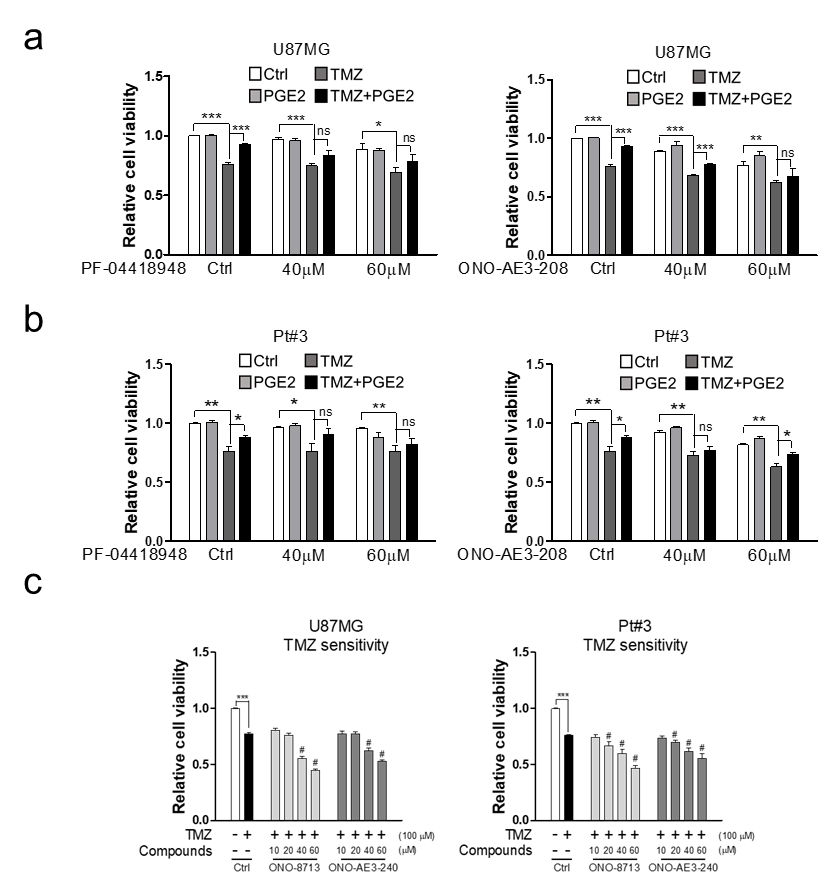


**Supplementary Figure S7. The effect of PGE2 receptor antagonists on TMZ sensitivity. (a)**, **(b)** U87MG cells were treated with EP2 antagonist PF-04418948 or EP4 antagonist ONO-AE3-208 in the presence of TMZ and PGE2 for 96 h, and cell viability was determined by MTT assay. Data were analyzed by two-tailed unpaired Student’s t test (n=3). **(c)** U87MG and Pt#3 cells were treated with different compounds in the presence of TMZ for 96 h, and cell viability was determined by MTT assay. Data were analyzed by two-tailed unpaired Student’s t test (n=3). (# means a significant difference with TMZ only).


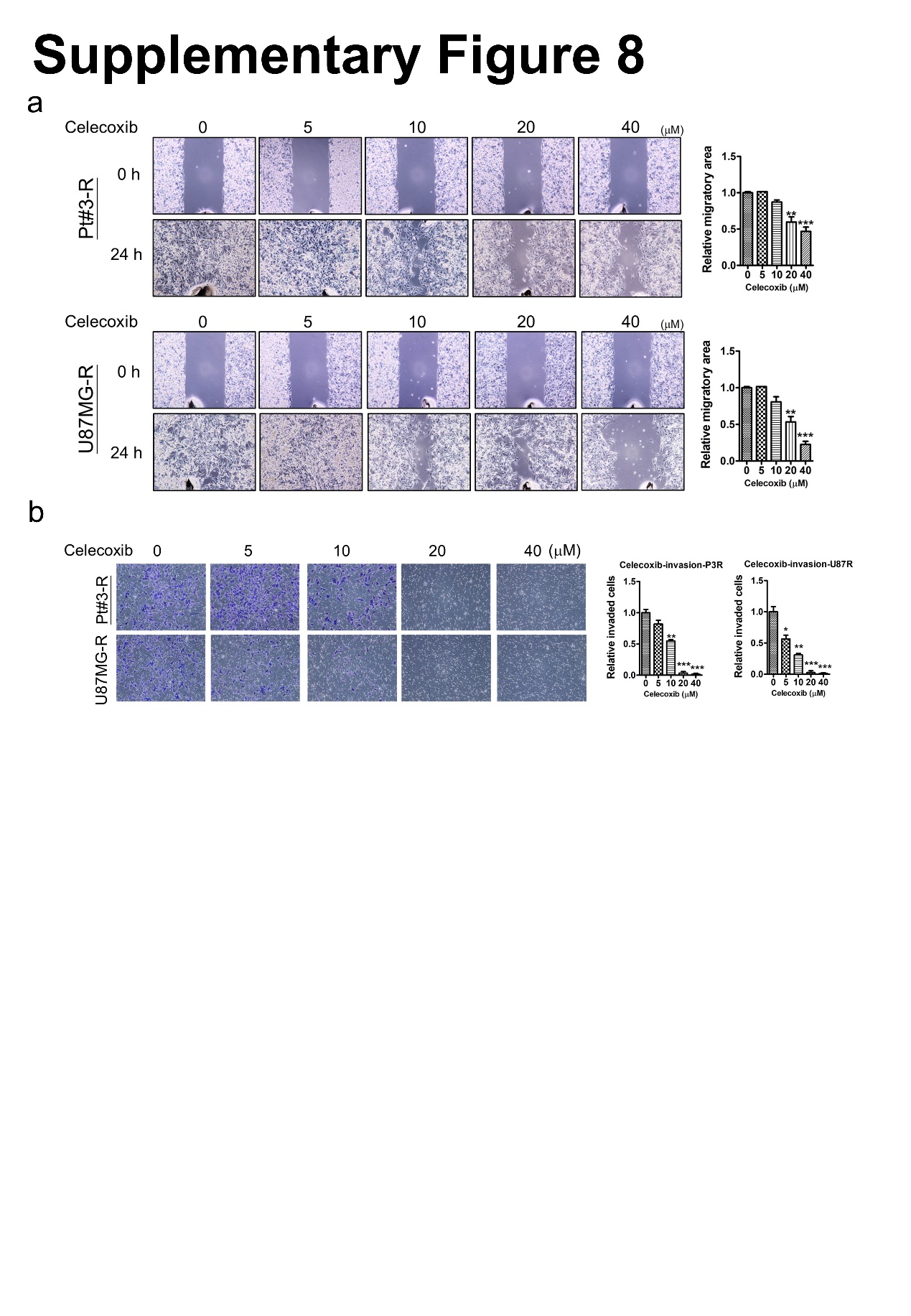


**Supplementary Figure S8. The effect of Celecoxib on TMZ-resistant GBM cell migration and invasion.** After treatment for 48 h, migratory and invasive activities of TMZ-resistant GBM cells were determined by wound-healing **(a)** and transwell invasion assays **(b)**, respectively.


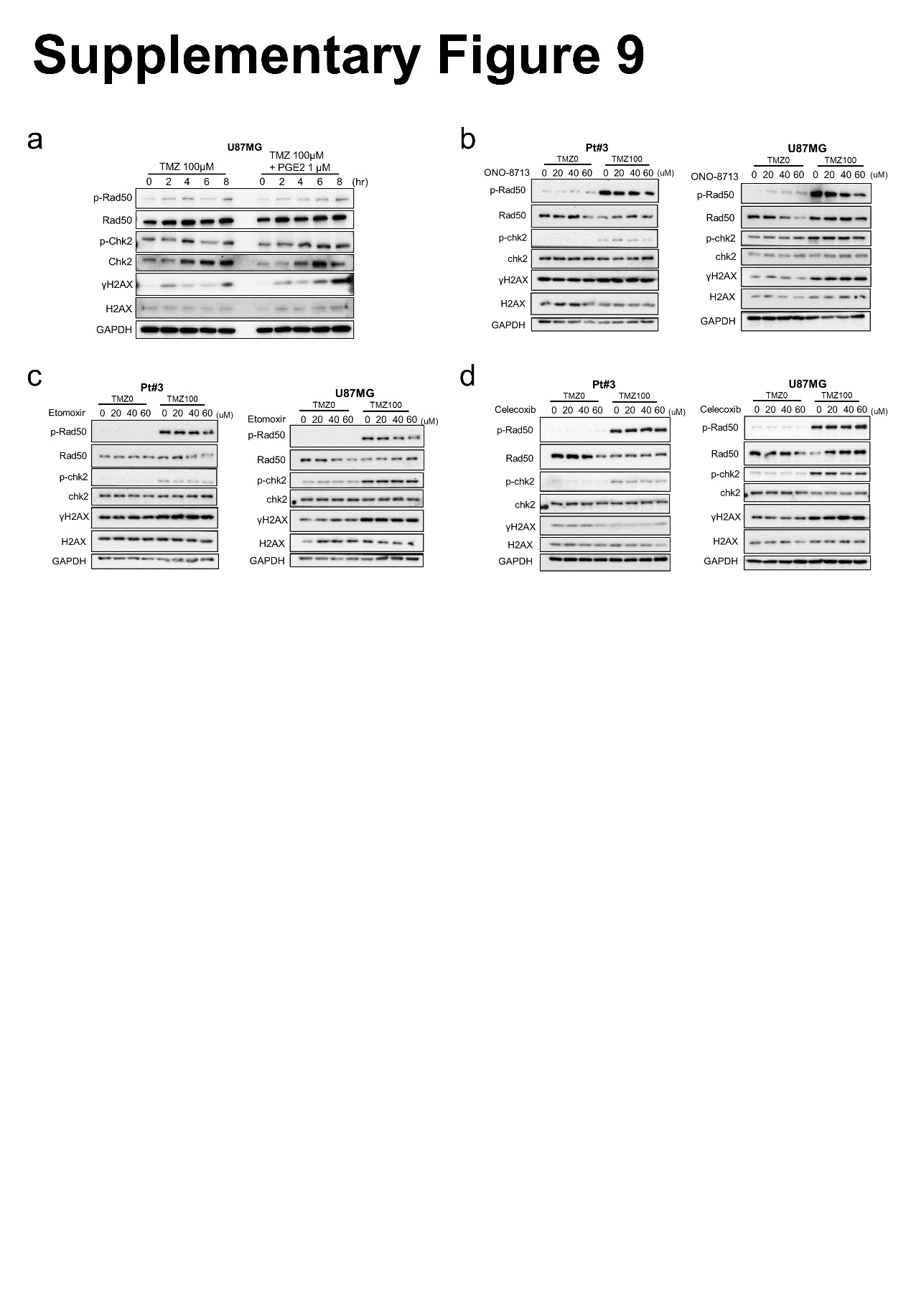


**Supplementary Figure S9. The DNA damage effect of PGE2, ONO-8713, Etomoxir, and Celecoxib with or without TMZ on GBM cells.** GBM Cells were treated with **(a)** PGE2, **(b)**ONO8713, **(c)** Etomoxir, and **(d)** Celecoxib with or without TMZ for 2 days. Cells then lysed to perform the Western blotting by using indicated antibodies.

**Supplementary Table S1. Characteristic of 14 GBM patients with recurrence involved in RNA-Seq analysis**

| **Characteristics** | **GBM001** | **GBM002** | **GBM003** | **GBM005** | **GBM006** | **GBM007** | **GBM009** |
| --- | --- | --- | --- | --- | --- | --- | --- |
| **Age at diagnosis** | 41 | 59 | 57 | 56 | 47 | 70 | 65 |
| **Gender** | Female | Female | Male | Female | Male | Male | Male |
| **Primary**  **pathology** | WHO grade Ⅲ  Anaplastic oligodendroglioma | WHO grade Ⅲ  Anaplastic oligodendroglioma | WHO grade Ⅳ  GBM | WHO grade Ⅳ  GBM | WHO grade Ⅳ  GBM | WHO grade Ⅳ  GBM | WHO grade Ⅳ  GBM |
| **Recurrent**  **pathology** | WHO grade Ⅲ  Anaplastic oligodendroglioma | WHO grade Ⅲ  Anaplastic oligodendroglioma | WHO grade Ⅳ  GBM | WHO grade Ⅳ  GBM | WHO grade Ⅳ  GBM | WHO grade Ⅳ  GBM | WHO grade Ⅳ  GBM |
| **Month without recurrence** | 43 | 54 | 26 | 20 | 15 | 21 | 32 |
| **IDH-1** | Negative | Positive | Wild type | Negative | Negative | Negative | No description |
| **GFAP** | Positive | Negative | Positive | Positive | Positive | Positive | No description |
| **P53** | No description | No description | Negative | No description | No description | No description | No description |
| **MGMT** | Negative | Negative | Negative | No description | No description | Positive | No description |

**Supplementary Table S1 -continued. Characteristic of 14 GBM patients with recurrence involved in RNA-Seq analysis**

| **Characteristics** | **GBM011** | **GBM012** | **GBM013** | **GBM014** | **GBM015** | **GBM016** | **GBM017** |
| --- | --- | --- | --- | --- | --- | --- | --- |
| **Age at diagnosis** | 35 | 49 | 54 | 66 | 32 | 47 | 60 |
| **Gender** | Male | Male | Male | Female | Male | Male | Male |
| **Primary**  **pathology** | WHO grade Ⅳ  GBM | WHO grade Ⅳ  GBM | WHO grade Ⅳ  GBM | WHO grade Ⅳ  GBM | WHO grade Ⅳ  GBM | WHO grade Ⅳ  GBM | WHO grade Ⅳ  GBM |
| **Recurrent**  **pathology** | WHO grade Ⅳ  GBM | WHO grade Ⅳ  GBM | WHO grade Ⅳ  GBM | WHO grade Ⅳ  GBM | WHO grade Ⅳ  GBM | WHO grade Ⅳ  GBM | WHO grade Ⅳ  GBM |
| **Month without recurrence** | 10 | 19 | 48 | 33 | 23 | 10 | 11 |
| **IDH-1** | No description | Negative | Negative | Wild type | Positive | Negative | Negative |
| **GFAP** | Positive | Positive | Positive | Positive | Positive | Positive | Positive |
| **P53** | No description | No description | No description | Negative | No description | No description | No description |
| **MGMT** | No description | No description | Negative | Negative | No description | Positive | No description |

**Supplementary Table S2. Characteristic of 5 GBM patients with recurrence involved in ω-3/6 fatty acid targeted metabolomics analysis**

| **Characteristics** | **Patient 1** | **Patient 2** | **Patient 3** | **Patient 4** | **Patient 5** |
| --- | --- | --- | --- | --- | --- |
| **Age at diagnosis** | 32 | 33 | 75 | 26 | 35 |
| **Gender** | Female | Male | Female | Female | Male |
| **Primary**  **pathology** | WHO grade Ⅳ  GBM | WHO grade Ⅳ  GBM | WHO grade Ⅳ  GBM | WHO grade Ⅱ  Diffuse astrocytoma | WHO grade Ⅱ  Diffuse astrocytoma |
| **Recurrent**  **pathology** | WHO grade Ⅳ  GBM | WHO grade Ⅳ  GBM | WHO grade Ⅳ  GBM | WHO grade Ⅲ  Anaplastic astrocytoma | WHO grade Ⅳ  GBM |
| **Month without recurrence** | 12 | 4 | 16 | 36 | 29 |
| **GBM-PNET^A^** |  | YES |  |  |  |
| **IDH-1** | Mutant | Mutant | Wild type | Mutant | Mutant |
| **GFAP** | Positive | Positive | No description | Positive | Positive |
| **P53** | Positive | No description | No description | Mutant | Mutant |
| **MGMT** | No description | Positive | No description | Positive | Negative |
| **PGE2** | Significant | Significant | Significant | Slight | Significant |

**Supplementary Table S3. The chemicals used in this study**

| Name | Cat. number | Company |
| --- | --- | --- |
| Temozolomide (TMZ) | T2577 | Sigma-Aldrich, St. Louis, MO, USA |
| Prostaglandin E2 (PGE2) | 14010 | Cayman, Ann Arbor, MI, USA |
| 16,16-dimethyl PGE2 (dmPGE2) | 14750 | Cayman, Ann Arbor, MI, USA |
| Prostaglandin D2 (PGD2) | 12010 | Cayman, Ann Arbor, MI, USA |
| Thromboxane B2 (TXB2) | 19030 | Cayman, Ann Arbor, MI, USA |
| Etomoxir | 11969 | Cayman, Ann Arbor, MI, USA |
| Saponin | SAE0073 | Sigma-Aldrich, St. Louis, MO, USA |
| Celecoxib | 10008672 | Cayman, Ann Arbor, MI, USA |
| Pyrrophenone | 13294 | Cayman, Ann Arbor, MI, USA |
| Zileuton | 10006967 | Cayman, Ann Arbor, MI, USA |
| ML-355 | 18537 | Cayman, Ann Arbor, MI, USA |
| ONO-8713 (EP1 antagonist) |  | Ono Pharmaceutical, Osaka, Japan |
| PF-04418948 (EP2 antagonist) | 15016 | Cayman, Ann Arbor, MI, USA |
| ONO-AE3-240 (EP3 antagonist) |  | Ono Pharmaceutical, Osaka, Japan |
| ONO-AE3-208 (EP4 antagonist) |  | Ono Pharmaceutical, Osaka, Japan |

**Supplementary Table S4. The information of plasmids in this study**

1. PLA2G5
2. Sequence: Start from 5’ to 3’

AATGACTGGTAACGTTGAAAATGTGCATGCCCCTCATCTGACCTATGCAATATGCTTACAAAAAACATTTAACCTGATAATCATGAGGAAGCAATAGGCAAATCCAAATTGGTGAGTATTCTGCAAGACAACTGGTCTGGGTTCAAGAGCATCAATGTCATTTTAAAGAAGAAACCAGATTAAGTCTGGCCGTGGTGGCTCACACCTGTAATTCCAGCACTTTGGAAGGCTGAGGCAGGTGGATCATTTGAACTCAGGAGTTTGAGACCAGACTGGGCAATATGGTGAAACCCCTTTCCACAAAAAATACAAAAAATTAGCCAGGCATGGGGTGGCATGTGCCTGTGGTCCCAGCTACTGGGAGGATCGCTGAAGCCCAGGAAGCCGAGGCTGCAGTGAGCTGAGATTGCACCACTGCACTCCAGCCTGGGTGACAGAGTGAGACCTCAGCTCAGAAAAGAAAGAAAGAAAGAAAGAAAGAAAGAAAGAAAGAAAGAAAGAAAGAAAGAAACCAGATTAAAGAACTTTCTAGATTAAAGGACACCAAAGAGCCACAACAATTAGATGCAATGCCTGATTCTTGTTGGGATTCTGGATCGGAAAAAAGCCAACAAATTGGATATAAAAACCATTATTGGGACAATAGGGGAAATTTGTATATTAGATAGTTTTACTGTATACTAGATACAGTATTGTGTTCATGTTCTTGTCCCCGCCAAGACTGATGAGCCCCCAGGGACTCAGATAGTCAAGATTCTAAGCAGGCTGTCAGAGCCTTAACAAGGTTCTGAGCCCAGGACTGAGGTAGGGGTTACTTCGGGCTGAGGGGATTAGCAAAGGCTTGTGAAGTGGCTAAAGCCGTGACCTGGATATTTCAGGACTCAAATCCACCCGCATTGGAATTCTCTGGGTGACTGCCCAGCCCCTTTGGCAGGACAGCCAGCCACAGGCCCTGATTGTGCAGGACTTCCTGCCTCTGCAAAGGCAGTCGGGGGCTGAG

1. Cutting enzyme: XhoI / HindIII
2. Primers: Start from 5’ to 3’

Forward: GTATCTCGAGAATGACTGGTAACGTTGAAA

Reverse: AGAAAGCTTCTCAGCGCCCGACTGCCTTT

**Supplementary Table S4 -continued. The information of plasmids in this study**

1. ABHD8
2. Sequence: Start from 5’ to 3’

TAGCAAAACATCACCACTATAATAATGCCCATAATATATGGAGCATTTGTTATATGCACGACACTATGATGAGTGCTTTACCTGCGTAGCAAACTTATGAGAGGATTAATTTCATAGAGAAATAAACGGAGGCTCAGGAAAGTGAAGTAACTTGCCCGAGGTCACACATCAGCAAAGGGCAGAGGCTGGATTTGAGACCTGAGCTCTCCTGAGGGCCTACCATGGCCCCGCCGGTACCACAGAGCTGGGATGCCTGCCTCTTGCTATCAGCCCTTCTCAGAACCATGGCAGCCATGGGTTAAATCGGCTGATTATGAAATTCAAACTCTCTAATTACCCACAGCTCCAAGACTTTCCTTGAAAGCTTTAGAGAAGCCCGTCACGATGGCTCACGCCAGTGATCTCAGCCCTTTGGGAGGCCGTAGACGAGAGGATTGCTTGATGCCCAGAGTTCGAGACCAGTCTGGGCAAGATAGCGAGACCTCGTCCCTACCAAAAGAAAAGGGGGGGGCGTAGGGGGGAGGTTAGCAAATAATGGTTCCCAGGGTTTGGAATCACACAGCCTTGGGTTCAAGTCCCAGCTTTGCTCCTTGCTTGATTTATGCTCTTGTGCCCTTTATGAGCCTCAGTTTCCCCACCGGAGATTGGAAAAAGATCTTGGAGTCTCTGCTTCTAGAATGGAAAGGTCAATACAGAGAGAAGAGTATGGGGGCCTCGTTTGGAGAGGAACAGGGCAATTGGGAGGCAGGGGCTGGGGGGCAAGGGCTTGGGAGACCCATTTGCAGAGAGAGGGCCCCGGCCGCACACTCCCAGGCGTCCCGCAGACCCTAGACTGCAACCCCCTTCTCCGCCTTGAGCCAGGCCCCATCCCTCCCCCAACCTCGGTGAACCCCCAAAAGGGGCGGGGCCGTTGCTCCCAGAGCCCGCCTCCACTACGGAATTGCCGCCTCTCATTGGGCTCCTAACTAGGCCAGGTCAGTCCGCAGCTGCGATAGGCCGA

1. Cutting enzyme: SmaI / HindIII
2. Primers: Start from 5’ to 3’

Forward: AGCCCCGGGTAGCAAAACATCACCACTAT

Reverse: GGCGCTCGAGTCGGCCTATCGCAGCTGCGG

**Supplementary Table S4 -continued. The information of plasmids in this study**

1. PTGS2
2. Sequence: Start from 5’ to 3’

AGAAGGCAGGAAACTTTATATTGGTGACCCGTGGAGCTCACATTAACTATTTACAGGGTAACTGCTTAGGACCAGTATTATGAGGAGAATTTACCTTTCCCGCCTCTCTTTCCAAGAAACAAGGAGGGGGTGAAGGTACGGAGAACAGTATTTCTTCTGTTGAAAGCAACTTAGCTACAAAGATAAATTACAGCTATGTACACTGAAGGTAGCTATTTCATTCCACAAAATAAGAGTTTTTTAAAAAGCTATGTATGTATGTGCTGCATATAGAGCAGATATACAGCCTATTAAGCGTCGTCACTAAAACATAAAACATGTCAGCCTTTCTTAACCTTACTCGCCCCAGTCTGTCCCGACGTGACTTCCTCGACCCTCTAAAGACGTACAGACCAGACACGGCGGCGGCGGCGGGAGAGGGGATTCCCTGCGCCCCCGGACCTCAGGGCCGCTCAGATTCCTGGAGAGGAAGCCAAGTGTCCTTCTGCCCTCCCCCGGTATCCCATCCAAGGCGATCAGTCCAGAACTGGCTCTCGGAAGCGCTCGGGCAAAGACTGCGAAGAAGAAAAGACATCTGGCGGAAACCTGTGCGCCTGGGGCGGTGGAACTCGGGGAGGAGAGGGAGGGATCAGACAGGAGAGTGGGGACTACCCCCTCTGCTCCCAAATTGGGGCAGCTTCCTGGGTTTCCGATTTTCTCATTTCCGTGGGTAAAAAACCCTGCCCCCACCGGGCTTACGCAATTTTTTTAAGGGGAGAGGAGGGAAAAATTTGTGGGGGGTACGAAAAGGCGGAAAGAAACAGTCATTTCGTCACATGGGCTTGGTTTTCAGTCTTATAAAAAGGAAGGTTCTCTCGGTTAGCGACCAATTGTCATACGACTTGCAGTGAGCGTCAGGAGCACGTCCAGGAACTCCTCAGCAGCGCCTCCTTCAGCTCCACAGCCAGACGCCCTCAGACAGCAAAGCCTACCCCCGCGCCGCGCCCTGCCCGCCGCTGCG

1. Cutting enzyme: XhoI / HindIII
2. Primers: Start from 5’ to 3’

Forward: TTTGCTCGAGAGAAGGCAGGAAACTTTATA

Reverse: GGCAAGCTTCGCAGCGGCGGGCAGGGCGC

**Supplementary Table S5. The antibodies used in this study**

| Antibody | Cat. Number | Company | Dilution |
| --- | --- | --- | --- |
| ACAA2 | Ab128911 | Abcam, Cambridge, UK | 1:1000 |
| CPT1A | A5307 | ABclonal, Woburn, MA, USA | 1:1000 |
| GAPDH | 60004-1-lg | Proteintech Group, Chicago, IL, USA | 1:1000 |
| GFP | 632592 | Takara Clontech Laboratories, Inc., CA, USA | 1:5000 |
| LDHA | GTX101416 | GeneTex, Inc., Irvine, CA, USA | 1:1000 |
| MFN1 | A9880 | ABclonal, Woburn, MA, USA | 1:1000 |
| MFN2 | GTX134774 | GeneTex, Inc., Irvine, CA, USA | 1:1000 |
| NQO1 | GTX113336 | GeneTex, Inc., Irvine, CA, USA | 1:1000 |
| OPA1 | GTX129917 | GeneTex, Inc., Irvine, CA, USA | 1:1000 |
| PTGES2 | A13440 | ABclonal, Woburn, MA, USA | 1:1000 |
| PTGS2 | A1253 | ABclonal, Woburn, MA, USA | 1:1000 |
| α-Tubulin | 66031-1-lg | Proteintech Group, Chicago, IL, USA | 1:5000 |
| β-Actin | 66009-1-lg | Proteintech Group, Chicago, IL, USA | 1:5000 |
| p-Rad50 | #14223 | Cell Signaling Technology, Inc., Beverly, MA, USA | 1:1000 |
| Rad50 | #3427 | Cell Signaling Technology, Inc., Beverly, MA, USA | 1:1000 |
| p-Chk2 | #2197 | Cell Signaling Technology, Inc., Beverly, MA, USA | 1:1000 |
| Chk2 | #6334 | Cell Signaling Technology, Inc., Beverly, MA, USA | 1:1000 |
| γH2AX | #9718 | Cell Signaling Technology, Inc., Beverly, MA, USA | 1:1000 |
| H2AX | #7631 | Cell Signaling Technology, Inc., Beverly, MA, USA | 1:1000 |

**Supplementary Table S6. The PCR primers (start from 5’ to 3’) used in this study**

| ACAA2 | Forward: AAA ACC AAT GTG AAT GGA GGA G |
| --- | --- |
|  | Reverse: GGC TGT GCT CTG AAT GAT GAC |
| COA7 | Forward: TAT CGG CTG GTG GAC TAT TTG |
|  | Reverse: TTG ACT TCT TTC CAG GCT TCT C |
| COX11 | Forward: TGG AAC TTT AGA CCT CAG CAA AC |
|  | Reverse: GAA GCC TGG AAG ACA ATA CCT G |
| CPT1A | Forward: CTT TCA GTT CAC GGT CAC TCC |
|  | Reverse: CAC CAC CAC GAT AAG CCA AC |
| MTHFD2L | Forward: TGG AAA CAG AAG ACC TCA CCT C |
|  | Reverse: CAT TGC ATA TTG TTC GCT CAT C |
| NDUFAF2 | Forward: GGT TGG TCT CAG GAT TTG TTC |
|  | Reverse: CCA TAG TAG GTG GAG TCT TTC TTG |
| PPA1 | Forward: CCT GAT GGA AAA CCA GAA AAT G |
|  | Reverse: ATC CAC GTC TGT TGG TAC TGT G |
| PTGS2 | Forward: TAC CCT CCT CAA GTC CCT GA |
|  | Reverse: ACT GCT CAT CAC CCC ATT CA |
| PTGE2S | Forward: CTC ATC AGC AAG CGA CTC AA |
|  | Reverse: GTG TGC TGC ATC AGG TCA TC |
